# Resected Brain Tissue, Seizure Onset Zone and Quantitative EEG Measures: Towards Prediction of Post-Surgical Seizure Control

**DOI:** 10.1371/journal.pone.0141023

**Published:** 2015-10-29

**Authors:** Christian Rummel, Eugenio Abela, Ralph G. Andrzejak, Martinus Hauf, Claudio Pollo, Markus Müller, Christian Weisstanner, Roland Wiest, Kaspar Schindler

**Affiliations:** 1 Support Center for Advanced Neuroimaging (SCAN), University Institute for Diagnostic and Interventional Neuroradiology, Inselspital, Bern, Switzerland; 2 Department of Neurology, Inselspital, Bern, Switzerland; 3 Universitat Pompeu Fabra, Department of Information and Communication Technologies, Barcelona, Spain; 4 Bethesda Epilepsy Clinic, Tschugg, Switzerland; 5 Department of Neurosurgery, Inselspital, Bern, Switzerland; 6 Centro de Investigaciones en Ciencias, Universidad Autónoma del Estado de Morelos, Cuernavaca, Mexico; 7 Centro Internacional de Ciencias, Universidad Autónoma de México, Cuernavaca, Mexico; University of Minnesota, UNITED STATES

## Abstract

**Background:**

Epilepsy surgery is a potentially curative treatment option for pharmacoresistent patients. If non-invasive methods alone do not allow to delineate the epileptogenic brain areas the surgical candidates undergo long-term monitoring with intracranial EEG. Visual EEG analysis is then used to identify the seizure onset zone for targeted resection as a standard procedure.

**Methods:**

Despite of its great potential to assess the epileptogenicty of brain tissue, quantitative EEG analysis has not yet found its way into routine clinical practice. To demonstrate that quantitative EEG may yield clinically highly relevant information we retrospectively investigated how post-operative seizure control is associated with four selected EEG measures evaluated in the resected brain tissue and the seizure onset zone. Importantly, the exact spatial location of the intracranial electrodes was determined by coregistration of pre-operative MRI and post-implantation CT and coregistration with post-resection MRI was used to delineate the extent of tissue resection. Using data-driven thresholding, quantitative EEG results were separated into normally contributing and salient channels.

**Results:**

In patients with favorable post-surgical seizure control a significantly larger fraction of salient channels in three of the four quantitative EEG measures was resected than in patients with unfavorable outcome in terms of seizure control (median over the whole peri-ictal recordings). The same statistics revealed no association with post-operative seizure control when EEG channels contributing to the seizure onset zone were studied.

**Conclusions:**

We conclude that quantitative EEG measures provide clinically relevant and objective markers of target tissue, which may be used to optimize epilepsy surgery. The finding that differentiation between favorable and unfavorable outcome was better for the fraction of salient values in the resected brain tissue than in the seizure onset zone is consistent with growing evidence that spatially extended networks might be more relevant for seizure generation, evolution and termination than a single highly localized brain region (i.e. a “focus”) where seizures start.

## Introduction

One third of patients suffering from focal epilepsies continue to have seizures despite of optimal medical treatment [1–3]. In the case of pharmacoresistant epilepsies, the selective resection of epileptogenic tissue considerably improves seizure control. Recent longitudinal trials indicated that long-term seizure freedom can be achieved in up to 2/3 of patients who undergo surgery [4–7].

Accurate localization of epileptogenic tissue is crucial for post-surgical seizure control. An important practical challenge is that with pre-surgical intracranial electroencephalography (iEEG)–or any other current diagnostic method–the brain tissue of the so-called “epileptogenic zone” (EZ), i.e. neuroanatomical areas that are necessary and sufficient to generate epileptic seizures, cannot be mapped directly and completely. Therefore, in clinical practice, the seizure onset zone (SOZ, i.e. the area where the first ictal EEG signal changes are recorded), is used as a proxy for the EZ [8]. However, given the limited spatial sampling of intracranial EEG recordings, the exact boundaries of the SOZ and the extent of overlap with the EZ remain unknown. Furthermore, the definition of the eventually resected brain tissue (RBT) depends not only on the localization and extent of the SOZ, but also on surrounding eloquent cortex and on the selected neurosurgical procedure. Thus, the question if a critical portion of the targeted epileptogenic network has been resected, is subject to post-hoc analysis of the post-surgical structural MRI: if a patient achieves long-term seizure freedom after epilepsy surgery, critical parts (or critical “nodes”, following network terminology) of the SOZ and/or EZ must have been included in the RBT.

To date, the clinical interpretation of iEEG recordings is mostly based on expert visual analysis, which is time-consuming and may yield a considerable degree of inter-rater variability. In the past decades, quantitative EEG (qEEG) analysis methods have been developed to identify epilepsy-related signals [9–14]. qEEG is more objective than visual analysis and may reveal subtle signal features and dynamics that are difficult or impossible to detect by visual inspection. Moreover, some qEEG measures are very sensitive to highly localized changes of intracranial EEG signals, and might thus help identify the critical parts/nodes of an epileptogenic network with high accuracy. To achieve better seizure control or even seizure freedom after surgery the EZ has to be delineated as precisely and objectively as possible. Therefore, qEEG has been recently invoked specifically to assess the brain areas targeted for surgical removal, see e.g. [15–22]. Some studies have explicitly addressed the overlap between channels highlighted by qEEG measures with the RBT [23–25]. Others have correlated resection of these areas with post-surgical seizure control [26–30].

Using inter-ictal iEEG recordings of five epilepsy patients, Andrzejak et al. [31] have recently shown that EEG signals derived from the epileptogenic cortex are less random, more nonlinear-dependent and more stationary than those recorded from non-focal brain regions. Previously, similar findings have been demonstrated for the hemispheric, but not sub-lobar level [32–34]. However, despite being precondition for SOZ localization, a difference in average qEEG measures between epileptogenic and non-epileptogenic (or focal/non-focal or ipsilateral/contralateral to the SOZ) cortex alone is not sufficient for precise delineation of candidate tissue for surgical resection. For practical diagnostic applications the overlap of distributions of qEEG measures between epileptogenic and non-epileptogenic brain tissue is crucial. In the present study we set out to investigate the extent to which the prevalence of saliency of certain iEEG channels with respect to four qEEG measures at a given time was associated with resection of the corresponding brain tissue and with surgery outcome in terms of seizure control. The selected qEEG measures have previously been used in the Bern group for iEEG analysis and are representative of four different main categories of signal analysis without making claims for completeness: the absolute signal slope S, the number N of forbidden ordinal patterns, a surrogate corrected cross-correlation matrix C and a surrogate corrected mutual information matrix M.

The absolute value S of the first temporal derivative (“slope”) of the signals represents a simple linear and univariate measure to quantify epileptiform EEG signals [35] and is–except for a scaling factor–identical to the “line length” feature [36]. The absolute slope S has for example been used to define objectively the onset and termination of seizures [37,38] as well as the spatial extent of secondary seizure generalization [39,40]. The so-called number (or fraction) of forbidden ordinal patterns N [41], another univariate but nonlinear qEEG measure, quantifies the degree of signal determinism. For qEEG analysis it was used in [42–44].

qEEG measures do not only allow to quantify features of individual signals (univariate measures), but also help to assess directed or non-directed signal interrelations and dependences (bivariate measures) and network properties (multivariate measures). As a non-directed, linear, bivariate qEEG measure we here use the “cross-correlation strength” matrix C [45,46] as a corrected version of Pearson's correlation matrix. This correction accounts for random effects due to the relative power in low frequencies and the limited duration of the moving window used for time-resolved signal analysis. A nonlinear, mutual information based analog M of the matrix C, where linear univariate properties as well as the effects of linear signal interrelations are compensated for, was introduced by Rummel et al. [46]. Here we apply the matrices C and M to a larger set of iEEG recordings for the first time.

The aim of our study is to test whether locally salient (“focal”) EEG features as detected by the four qEEG measures X = {S,N,C,M} at any time step can be used to identify brain parts that should be included within the RBT to achieve seizure freedom. To this end, we compare the dynamics of the qEEG measures X immediately before, during and directly after seizures in patients with focal epilepsy syndromes. Specifically, focal EEG features that stand out from the background are defined in a dynamic and data-driven manner from the distribution of X across iEEG channels at each time step. To link the qEEG results with clinical findings we assess not only the sub-group of patients with favorable post-surgical outcome but as a contrast also unfavorable outcomes. An important aspect of our study is that by using image coregistration procedures, the anatomical positions of iEEG contacts are precisely localized and the corresponding iEEG signals are categorized into the zones Z = {RBT,SOZ,OVL,NON}. Here, OVL is defined as the overlap of RBT and SOZ. These channels also contribute to the RBT and the SOZ individually. The zone NON comprises all channels that neither recorded from the RBT nor from the SOZ.

We find that the four qEEG measures X reveal different properties in the different zones Z. For S, N and M salient values are overrepresented in the RBT of patients with post-surgical seizure control. In contrast, in those patients who have not been rendered seizure-free post-surgically, salient values are often localized outside the RBT. We conclude that part of the investigated qEEG measures provide clinically relevant and objective markers of target tissue for optimizing epilepsy surgery.

## Materials and Methods

### Patients

We included 16 patients of the epilepsy surgery program of the Inselspital Bern in this study (11 female, 5 male; median age 31.0y, IQR 15.3y, range 19-59y). Demographic data on the selected patients is compiled in Table 1. Six patients fell into Engel class I (free of disabling seizures), five into Engel class II (rare disabling seizures) and five into Engel class IV (no worthwhile improvement). Inclusion criteria were that post-surgical follow up was available for at least one year (median 3.0y, IQR 1.3y) and high resolution pre- and post-operative T1-weighted (T1w) MRI had been registered. To avoid introducing a bias towards patients with the largest number of seizures during intracranial EEG monitoring (median 4.5, IQR 5.5, range 2–14), only two seizures of each patient were included in the study (five patients from all Engel classes had only two seizures during long-term monitoring with iEEG, see Table 1). If a patient had several seizure types (pat. I-5, IV-3 and IV-4), two seizures of each type were included. For each seizure type we selected the first two seizures occurring during iEEG monitoring for qEEG analysis.

**Table 1:** Demographic and clinical characteristics.

age (y)	syn-drome	hemi-sphere	lesion MRI visible	follow up (y)	No. of seizures during phase II	duration of phase II (d)	mean seizure frequency during phase II	No. of seizure types during phase II	No. of artifact free iEEG channels	total intracranial volume (ml)
26	MTLE	R	y (hippocampal sclerosis)	3	2	8	0,250	1	64	1437
48	MTLE	L	y (hippocampal sclerosis)	3	4	5	0,800	1	64	1297
27	LTLE	L	n	1	9	10	0,900	1	56	1321
36	PLE	L	y (pilocytic astrocytoma)	5	7	4	1,750	1	74	1627
19	MTLE	L	y (hippocampal sclerosis)	5	4	10	0,400	2	40	1426
25	FLE	R	n	4	2	11	0,182	1	99	1518
**26,5**				**3,5**	**4,0**	**9,0**	**0,600**	**1,0**	**64,0**	**1431**
**8,5**				**1,8**	**3,8**	**4,3**	**0,588**	**0,0**	**13,5**	**151**
49	FLE	R	y (focal cortical dysplasia)	4	6	9	0,667	1	92	1248
46	LTLE	R	n	3	5	8	0,625	1	100	1093
20	LTLE	R	n	3	10	5	2,000	1	54	1623
31	LTLE	L	y (hippocampal sclerosis)	3	6	8	0,750	1	59	1233
24	LTLE	L	n	3	2	6	0,333	1	47	1347
**31,0**				**3,0**	**6,0**	**8,0**	**0,667**	**1,0**	**59,0**	**1248**
**22,0**				**0,0**	**1,0**	**2,0**	**0,125**	**0,0**	**38,0**	**114**
38	LTLE	L	n	4	2	8	0,250	1	59	1430
23	LTLE	L	n	2	3	6	0,500	1	61	1248
59	MTLE	L	y (space occupying amygdala)	4	10	6	1,667	2	49	1286
32	PLE	L	y (focal cortical dysplasia)	2	14	6	2,333	2	96	1711
31	FLE	R	y (tuberous sclerosis)	2	2	5	0,400	1	32	1321
**32,0**				**2,0**	**3,0**	**6,0**	**0,500**	**1,0**	**59,0**	**1321**
**7,0**				**2,0**	**8,0**	**0,0**	**1,267**	**1,0**	**12,0**	**143**
**31,0**				**3,0**	**4,5**	**7,0**	**0,646**	**1,0**	**60,0**	**1334**
**15,3**				**1,3**	**5,5**	**2,5**	**0,708**	**0,0**	**25,8**	**180**
**0,672**	**0,799**	**0,744**	**0,868**	**0,532**	**0,811**	**0,5**	**0,855**	**0,265**	**0,721**	**0,295**

A 1^st^ level Kruskal-Wallis test was used to test the null hypothesis that all data stem from the same distribution, pair-wise 2^nd^ level Mann-Whitney-Wilcoxon tests were used to assess differences between the class-wise medians.

Abbreviations: F: female, M: male, FLE: frontal lobe epilepsy, LTLE: lateral temporal lobe epilepsy, MTLE: mesial temporal lobe epilepsy, PLE: parietal lobe epilepsy, L: left, R: right, MRI: magnetic resonance imaging, y/n: yes/no, n.a.: not applicable

This study was approved by the Internal Review Board of the Inselspital (approval No. 159399, dated 26^th^ of November, 2013). All patients gave written informed consent that imaging and EEG data may be used for research purposes. The decision on the necessity for intracranial EEG diagnostics, the chosen electrode implantation scheme and the decision on surgical epilepsy therapy was made entirely on clinical grounds. These decisions were taken prior to and independently from the present retrospective study.

### EEG acquisition

EEG signals were recorded intracranially by strip, grid and depth electrodes (all manufactured by AD-TECH, Wisconsin, USA), using a NicoletOneTM recording system with a C64 amplifier (VIASYS Healthcare Inc., Madison, Wisconsin, USA). An extracranial electrode, localized between 10–20 positions Fz and Cz, was used as reference for signal recording. EEG recordings were either sampled at 512 or 1024 Hz, depending on whether they were recorded with less or more than 64 contacts. The latter were down-sampled to 512 Hz prior to further analysis, and EEG signals were re-referenced against the median of all the channels free of permanent artifacts as judged by visual inspection. In addition to anti-aliasing filtering needed for proper sampling and down-sampling, EEG signals were digitally band-pass filtered between 0.5 and 150 Hz using a fourth-order Butterworth filter prior to analysis. Forward and backward filtering was applied to minimize phase distortions.

### Visual EEG analysis

Besides clinical EEG interpretation, for our study all recordings were visually inspected by an experienced epileptologist/electroencephalographer (K.S.) for four purposes: First, channels that were continuously corrupted by artifacts of any kind were identified and excluded from further analysis. The number of remaining iEEG channels is denoted as n (median 60.0, IQR 25.8, range 32–100, see Table 1). Second, the time points of seizure onset and termination were determined for all included seizures. From this information the following epochs were deduced: Three consecutive epochs of one minute duration immediately before seizure onset, three consecutive epochs of one minute duration immediately after seizure termination and six equally sized epochs during seizure evolution (three termed “early ictal” and three “late ictal”). Third, the iEEG channels were identified that recorded from the visually defined SOZ. Finally, the number of seizure types per patient was determined (different SOZ and/or different sequence of seizure states).

Seizure onsets were visually identified as the time of earliest EEG change associated with seizures following a systematic approach as previously described by Litt et al. [47]. We first identified unequivocal seizure activity and then moved backward in time to the moment when the first sustained change of EEG relative to the background pattern occurred. Repetitive high amplitude spike-and-wave signals or a flattening followed by low voltage fast activity [48] were the most frequently observed changes. Unequivocal seizure onset was defined as any epileptiform signal pattern, which became clearly identifiable without knowing that a seizure followed. Seizure termination on the other hand was identified as the time point when clearly identifiable epileptiform signals vanished, which typically manifested as a sudden drop of amplitude and frequency.

### Quantitative EEG analysis

Independent from visual EEG analysis for clinical purposes we analyzed peri-ictal epochs of the selected seizures with four qEEG measures. Quantitative signal analysis was done with C and MATLAB (MathWorks, Natick, MA., USA) programs developed by the authors.

#### Univariate linear qEEG measure: absolute signal slope S

The absolute value of the first temporal derivative of the signals (“slopes”) was used to detect epileptiform activity in single iEEG channels in an objective way, as previously described in [37–39]. In brief, the slope of the iEEG signals was estimated by the differences

$$dx_{i}\left(t\right)=\frac{x_{i}\left(t+\Delta t\right)-x_{i}\left(t\right)}{\Delta t}$$ (1)

where *i* and *t* denote the channel and the temporal sampling points and Δ*t* is the sampling interval, respectively. The absolute value S of the signal slope defined in Eq 1 provides an appropriate characterization of epileptiform EEG, since it is large for both slow, high amplitude signals as well as for fast, low amplitude signals [35]. The absolute signal slopes S were averaged over T = 1024 sample points (2 seconds) and normalized by dividing by their channel-wise standard deviation during a pre-ictal reference period of 60 seconds duration starting three minutes before visual seizure onset.

#### Univariate nonlinear qEEG measure: number of forbidden ordinal patterns N

We quantified signal determinism (as opposed to stochasticity) by the number of forbidden ordinal patterns N. To compute this quantity we followed an approach proposed by Amigó et al. [41] and recently applied to EEG data by [42–44]. A univariate iEEG signal *x*_*i*_(*t*) was mapped to a finite number of ordinal patterns by first choosing two parameters, a pattern order d>1 and a time delay τ≥1. Then d observations *x*_*i*_(*t*) spaced at τ sampling intervals of length Δ*t* were determined to generate an embedding vector of dimension d:

$${\vec{x}}_{i,t}=\left(x_{i}(t-(d-1)\tau \Delta t),\ldots \;,x_{i}(t-\tau \Delta t),x_{i}(t)\right)$$ (2)

The elements of the vector ${\vec{x}}_{i,t}$ were mapped uniquely onto the permutation π = (π_0_, π_1_, …, π_d-1_) of (0, 1,…, d−1) that fulfilled

$$x_{i}\left(t-\pi_{0}\tau \Delta t\right)\leq x_{i}\left(t-\pi_{1}\tau \Delta t\right)\leq x_{i}\left(t-\pi_{d-1}\tau \Delta t\right)$$ (3)

Equal values were ordered according to the time of their appearance in ${\vec{x}}_{i,t}$. As an example, the 5-dimensional embedding vector ${\vec{x}}_{i,t}$ = (1.26, 6.38, 0.63, 1.26, 4.92) was mapped onto the ordinal pattern π = (2, 4, 1, 0, 3). In d dimensions we have d! different permutations π in total and the sample size of ordinal patterns generated with maximal overlap from a time series of length T is T−(d−1)τ. Useful inequalities for the parameters d, τ and T in relation to the power spectrum of the signals have been derived in [44]. In the present paper we chose d = 5, τ = 1 and T = 1024 sample points (2 seconds) for the calculation of N. Normalization was performed in the same way as for the absolute signal slope S using the same pre-ictal reference epoch.

#### Multivariate linear qEEG measure: surrogate corrected cross-correlation matrix C

Pearson's equal-time (zero-lag) cross-correlation coefficient ρ is a linear measure for the dependence of two time series (or data sets in general). For infinitely long and independent time series of arbitrary power spectrum, ρ equals zero. However, time series of finite length–as typically result when using a moving window approach for time-resolved EEG analysis–may give rise to rather large random values of ρ, even when the time series are completely independent. In particular, such spurious correlations can occur when low frequencies (with regard to the short duration of the observed time series) dominate the power spectrum.

To estimate the strength of this random correlation we generated a set of *n*_surr_ independent realizations of *univariate* iterated amplitude adjusted Fourier transform (IAAFT) surrogate time series [49]. In mathematical terms, the univariate IAAFT surrogates are used to estimate the width of the zero-centered distribution of Pearson coefficients ρ under the null hypothesis that (i) the time series are two independent stationary linear stochastic auto-correlated Gaussian processes. (ii) The measurement functions by which the signals were derived from the dynamics are invertible but potentially nonlinear. (iii) The auto-correlations, means, and variances of the underlying Gaussian processes are such that the measurements result in the auto-correlations and amplitude distributions of the observed time series.

For positive ρ a formula that compensates for random correlations can heuristically be written as [45,46]:

$$C=\frac{\rho -\langle \rho_{\text{surr}}\rangle }{1-\langle \rho_{\text{surr}}\rangle }s$$ (4)

Here, ρ is the Pearson coefficient of the original data and 〈*ρ*_surr_〉 is the median of the values obtained from the set of surrogate time series. s is a significance factor that assumes the value one if the null hypothesis that 〈*ρ*_surr_〉 has larger absolute value or is equal to ρ can be rejected and zero otherwise. Details on the generalization of Eq 4 to negative ρ can be found in [45,46]. Surrogate based baseline correction strategies similar to Eq 4 were also followed in [31–34,50–54].

In the present study we followed a moving window approach to analyze the non-random correlation pattern of the entire peri-ictal iEEG recordings in time-resolved manner. To this end windows of 4096 sample points length (i.e. 8 seconds) were shifted over the recording with 512 sample points displacement (i.e. 1 second). On each time step the “genuine cross-correlation strength matrix" C [45,46] was constructed element-wise from Pearson's correlation matrix with coefficients ρ. For each window and EEG channel *n*_surr_ = 10 univariate IAAFT surrogates of *T*_surr_ = 4096 sampling points length were generated independently. In generalization of the heuristic formula Eq 4 the correlation coefficients ρ and *ρ*_surr_ were calculated from partially overlapping subsegments of T = 1024 sampling points length (i.e. 2 seconds). Within the windows these subsegments were distributed with minimal overlap to generate ensembles of size *n*_ens_ = 10 for the original time series and *n*_ens_ *n*_surr_ = 100 for the surrogates. A non-parametric Mann-Whitney-Wilcoxon U-test was performed to assess the significance of different medians of ρ and *ρ*_surr_ and to determine the significance factor s. To account for multiple comparisons (the n-dimensional correlation matrix has n*(n-1)/2 different channel combinations) Bonferroni corrections were applied independently on each time step. As a quantifier of the contributions of individual channels to the correlation pattern we calculated the node strength known from network analysis [55], i.e. the sum over the absolute values of all matrix elements connecting any iEEG channel with the other channels, and normalized it by dividing by the maximally possible value n-1.

#### Multivariate nonlinear qEEG measure: surrogate corrected mutual information matrix M

Mutual information μ is a model-free information theory based measure for interrelation and is in particular not restricted to Gaussianity or linear dependence. It quantifies the deviation of the observed joint distribution of signal amplitudes *x*_*i*_(*t*) and *x*_*j*_(*t*) from the product of the marginal distributions, which would imply statistical independence. In the present study mutual information between channel pairs was estimated from multivariate EEG time series *x*_*i*_(*t*) using the k-nearest-neighbor algorithm [56] with parameter k = 3 as implemented in the publicly available MILCA package (http://www.ucl.ac.uk/ion/departments/sobell/Research/RLemon/MILCA/MILCA). Especially for short (*T* ≤ 1000) and noisy time series this algorithm has been shown to be superior to other estimators [57]. Using a transformation suggested by Joe [58] μ can be normalized to the interval [0,1]. For positively correlated linear stochastic and Gaussian distributed data this transformation in addition warrants identity of μ with ρ. If the restrictions are relaxed, the Pearson coefficient represents a lower bound for normalized mutual information [59].

To correct for the influence of the linear ρ on the more general μ a very similar baseline correction as the one described in detail above was followed [46]. It differed only by using *multivariate* rather than univariate IAAFT surrogates [49] to sample the distribution of μ and by replacing ρ by μ and C by M in the heuristic formula Eq 4. Multivariate IAAFT surrogates resample the observed time series to test the following null hypothesis. (i) the dynamics is a stationary multivariate linear stochastic auto- and cross-correlated Gaussian process. (ii) The measurement functions by which the signals were derived from the dynamics are invertible but potentially nonlinear. (iii) The auto- and cross-correlations, means and variances of the underlying Gaussian process are such that the measurements result in the auto- and cross-correlations and amplitude distributions of the observed time series. For practical implementation of multivariate IAAFT surrogates we used the same segment lengths and sample size parameters as for the univariate IAAFT surrogates. Like for the linear matrix C we used Bonferroni correction to account for multiple comparisons and the normalized node strength as channel-wise quantifier of nonlinear interrelation.

### MR and CT image acquisition

All MRI scans were performed on a 3T Siemens Magnetom Trio (Erlangen, Germany) equipped with a 12-channel radio frequency head coil. High-resolution T1w MR images were obtained with a 3D Modified Driven Equilibrium Fourier Transform (MDEFT) [60] sequence. The optimized acquisition parameters included: 256 × 224 x 176 matrix points with a non-cubic field of view of 256 mm × 224 mm x 176 mm, yielding a nominal isotropic resolution of 1 mm^3^ (i.e. 1mm × 1mm × 1mm), repetition time TR = 7.92 ms, echo time TE = 2.48 ms, flip angle = 16°, inversion with symmetric timing (inversion time 910 ms), fat saturation, 12 minutes total acquisition time.

Within 24 hours after electrode implantation, every patient underwent a CT scan to control electrode localization and rule out subdural or intracranial hematoma. Following the safety protocols at our institution post-implantation MRI were not acquired. A follow up MRI using the same sequence and parameters as before electrode implantation was acquired three to four months after resective surgery.

#### Image processing and coregistration

First, T1w pre- and post-operative MR images as well as CT images were coregistered (without reslicing) to MNI-templates from each modality using SPM8 (http://www.fil.ion.ucl.ac.uk/spm/software/spm8/). The MNI-CT template was taken from the Clinical Toolbox [61] (http://www.nitrc.org/projects/clinicaltbx/). In a second step, CT and post-T1w images were coregistered to the pre-T1w image maximizing normalized mutual information [62], and resliced to 1 mm^3^ isotropic resolution. The pre-T1w image was then segmented (in native space) into tissue probability maps of grey matter (GM), white matter (WM) and cerebrospinal fluid (CSF) using VBM8 (http://dbm.neuro.uni-jena.de/vbm/). The segmentation routine contains an additional step to calculate the partial volume estimates (PVE) of the main GM, WM and CSF as well as mixed GM-WM and GM-CSF tissue classes, following methods described in [63]. A PVE image was thus generated where each voxel was labeled according to the tissue class it belongs to. This labeled PVE image was thresholded at a value of 1 to generate a binary mask of the brain and surrounding CSF (since parts of the iEEG electrodes are located in the subdural space). We then applied this mask to the coregistered CT images and thresholded the resulting image at a threshold of 2,500 Hounsfield units. This procedure segregated the intracranial electrodes into an “electrode only”-image, essentially using the CT-artifacts generated by the implanted electrodes as a proxy for their location. Finally, the RBT was identified in the post-T1w images and manually segmented using MRIcron (http://www.mccauslandcenter.sc.edu/mricro/mricron/), resulting in a binary RBT mask.

Three-dimensional renderings of the pre-T1w, electrode image and RBT mask were used in MRIcron to match each contact to the corresponding iEEG channel, and to determine their spatial relationship to the RBT. Channels recording from the RBT are indicated in red in all figures. Clinical reports of the intracranial recordings and previous visual analysis of the iEEG were used to identify iEEG channels belonging to the SOZ (blue in all figures). Channels that corresponded to the overlap (OVL) of RBT and SOZ are colored in magenta in all figures. Finally, 3D visualizations of the RBT, electrode image, and critical iEEG channels were generated using BrainNetViewer (http://www.nitrc.org/projects/bnv/) [64].

### Statistics

To analyze focal EEG features as quantified by the four qEEG measures X = {S,N,C,M} we separated channels with saliently high values from those yielding normal and small values on the same time step. The separation, i.e. the “saliency”, was obtained by determining the first and third quartile (Q_1_ and Q_3_) as well as the inter-quartile range IQR = Q_3_- Q_1_ of the distribution of X separately on each time step. All channels with values larger than Q_3_+w*IQR with w = 1.5 were defined as salient channels. The advantage of this dynamic and data-driven approach is that minimal assumptions have to be made about the shape of the underlying distributions, which may even change from time step to time step. Such an approach is well known from the definition of “outliers” at the upper end of box-and-whisker-plots and is applicable also for very skew distributions. In addition, w is the only parameter, for which we use the standard choice for outlier definition. Importantly, no assumption is made about the prevalence of salient values indicated by measure X = {S,N,C,M}. As they need to be larger than Q_3_, their number satisfies 0 ≤ *n*_*X*_ ≤ *n*/4 in a sample of size n and crucially depends on the shape of the distribution of X.

The fraction of focal salient EEG channels indicated by measure X found inside one of the channel zones of interest Z = {RBT,SOZ,OVL,NON} with size 0 ≤ *n*_*Z*_ ≤ *n* was calculated as the relative size of the channel intersection $F_{Z}^{X}\;=n_{Z\cap X}/n_{X}$. The values of $F_{Z}^{X}$ are dependent on the number of salient channels *n*_*X*_, which typically varies as a function of time in the peri-ictal evolution, as well as on the spatial extent *n*_*Z*_ of the zones Z, which varies from patient to patient (RBT) or may even vary from seizure to seizure (SOZ, OVL and NON). As both *n*_*X*_ and *n*_*Z*_ may independently become zero (and in consequence also *n*_*Z*∩*X*_ = 0), a simple normalization of $F_{Z}^{X}$ to the relative size *n*_*Z*_/*n* of zone Z may be ill defined. Thus, we complementarily assessed the significance of the fraction of salient values within zone Z using score statistics. The probability to find exactly *ν* salient values inside Z and *n*_*X*_ − *ν* outside Z (no replacements allowed) is given by the probability density function of the hypergeometric distribution [65]:

$$p_{n_{X},n_{Z}}\left(\nu \right)=\frac{\left(\begin{array}{c} n_{Z}\\ \nu \end{array}\right)\left(\begin{array}{c} n-n_{Z}\\ n_{X}-\nu \end{array}\right)}{\left(\begin{array}{c} n\\ n_{X} \end{array}\right)}$$ (5)

Values of *ν* are restricted to the interval between *ν*_min_ = max(0,*n*_*Z*_ + *n*_*X*_-n) and *ν*_max_ = min(*n*_*Z*_,*n*_*X*_), with a positive lower limit in conditions with more salient channels of measure X than channels outside zone Z (*n*_*X*_ > *n* – *n*_*Z*_). The cumulative probability of finding m or more salient values inside zone Z yields the significance of a hypergeometric test for over-representation:

$$P_{n_{X},n_{Z}}\left(m\right)=\sum_{\nu =m}^{\nu_{\text{max}}}\;p_{n_{X},n_{Z}}\left(\nu \right)$$ (6)

We report our results as the negative logarithms of Eq 6 with large values indicating high significance of the observation: $L_{Z}^{X}=-{\text{log}}_{10}\left(P_{n_{X},n_{Z}}\right)$.

All statistics were performed on significance level α = 0.05. As this is an explorative study in a small group of patients, no correction for multiple comparisons (four measures X, three zones Z, three Engel classes) was performed for group comparison. For comparison of categorial data (gender, syndrome etc.) the χ^2^-statistics was used. As the χ^2^-test is strictly applicable only if the smallest expected bin filling is five or larger, we resampled the group allocation randomly 10,000 times to estimate the p-values. To assess differences in the distribution of ordinal quantifiers (age, qEEG measures etc.) over several outcome classes the non-parametric Kruskal-Wallis test was applied. Conditional on the test result we performed post-hoc pair-wise comparisons with a Mann-Whitney-Wilcoxon U-test to check which classes indeed differed.

## Results

### Demographics and clinical information

Patient information is summarized in Tables 1 and 2. At a significance level of α = 0.05, none of the quantifiers differed between the three outcome classes. Trends were only observed towards different fraction of iEEG channels in the overlap OVL between of RBT and SOZ (p = 0.076) and in their union (p = 0.073).

**Table 2:** Characteristics of channel zones Z.

patient	RBT volume (ml)	RBT volume fraction	fraction of channels in RBT	fraction of channels in SOZ				fraction of channels in overlap OVL				fraction of channels in union of SOZ and RBT = 100% minus fraction of channels in NON				Jaccard index for overlap of SOZ and RBT			
				Sz1	Sz2	Sz1'	Sz2'	Sz1	Sz2	Sz1'	Sz2'	Sz1	Sz2	Sz1'	Sz2'	Sz1	Sz2	Sz1'	Sz2'
I-1	43,1	3,0%	31,3%	10,9%	10,9%			10,9%	10,9%			31,3%	31,3%			35,0%	35,0%		
I-2	20,6	1,6%	20,3%	17,2%	17,2%			14,1%	14,1%			23,4%	23,4%			60,0%	60,0%		
I-3	12,3	0,9%	8,9%	3,6%	3,6%			1,8%	1,8%			10,7%	10,7%			16,7%	16,7%		
I-4	19,8	1,2%	8,1%	1,4%	1,4%			1,4%	0,0%			8,1%	9,5%			16,7%	0,0%		
I-5	22,1	1,6%	27,5%	27,5%	50,0%	5,0%	10,0%	5,0%	15,0%	0,0%	5,0%	50,0%	62,5%	32,5%	32,5%	10,0%	24,0%	0,0%	15,4%
I-6	54,8	3,6%	11,1%	2,0%	2,0%			1,0%	1,0%			12,1%	12,1%			8,3%	8,3%		
**I: median**	**21,4**	**1,6%**	**15,7%**	**7,5%**				**3,4%**				**23,4%**				**16,7%**			
**I: IQR**	**17,9**	**1,3%**	**16,2%**	**13,2%**				**9,8%**				**21,2%**				**23,5%**			
II-1	8,5	0,7%	8,7%	4,3%	6,5%			2,2%	4,3%			10,9%	10,9%			20,0%	40,0%		
II-2	28,0	2,6%	13,0%	16,0%	12,0%			4,0%	5,0%			25,0%	20,0%			16,0%	25,0%		
II-3	33,5	2,1%	25,9%	13,0%	13,0%			3,7%	3,7%			35,2%	35,2%			10,5%	10,5%		
II-4	26,4	2,1%	28,8%	3,4%	3,4%			3,4%	3,4%			28,8%	28,8%			11,8%	11,8%		
II-5	32,8	2,4%	51,1%	25,5%	25,5%			12,8%	12,8%			63,8%	63,8%			20,0%	20,0%		
**II: median**	**28,0**	**2,1%**	**25,9%**	**12,5%**				**3,9%**				**28,8%**				**18,0%**			
**II: IQR**	**6,4**	**0,4%**	**15,8%**	**10,3%**				**1,4%**				**14,0%**				**8,2%**			
IV-1	17,6	1,2%	3,4%	6,8%	8,5%			0,0%	1,7%			10,2%	10,2%			0,0%	16,7%		
IV-2	23,7	1,9%	16,4%	21,3%	21,3%			4,9%	4,9%			32,8%	32,8%			15,0%	15,0%		
IV-3	21,7	1,7%	16,3%	14,3%	10,2%	8,2%	8,2%	6,1%	6,1%	0,0%	0,0%	24,5%	20,4%	24,5%	24,5%	24,9%	29,9%	0,0%	0,0%
IV-4	5,5	0,3%	4,2%	5,2%	5,2%	6,3%	6,3%	3,1%	4,2%	0,0%	0,0%	6,3%	5,2%	10,4%	10,4%	50,0%	80,0%	0,0%	0,0%
IV-5	12,2	0,9%	9,4%	15,6%	6,3%			0,0%	0,0%			25,0%	15,6%			0,0%	0,0%		
**IV: median**	**17,6**	**1,2%**	**9,4%**	**8,2%**				**0,8%**				**18,0%**				**7,5%**			
**IV: IQR**	**9,5**	**0,8%**	**12,2%**	**7,0%**				**4,7%**				**14,3%**				**22,8%**			
**median**	**21,9**	**1,6%**	**14,7%**	**8,3%**				**3,5%**				**24,0%**				**15,7%**			
**IQR**	**12,9**	**1,1%**	**17,4%**	**9,9%**				**4,0%**				**21,8%**				**15,3%**			
**p_1st**	**0,204**	**0,283**	**0,214**	**0,606**				**0,076**				**0,073**				**0,345**			

Statistics is the same as in Table 1.

Abbreviations: RBT: resected brain tissue, SOZ: seizure onset zone, OVL: overlap of RBT and SOZ, NON: channels neither in RBT nor in SOZ, n.a.: not applicable

### Peri-ictal qEEG analysis

**Fig 1:**
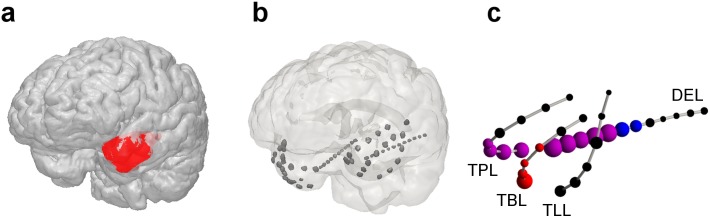
Neuroanatomical representation of resected brain tissue (RBT, panel a), intracranial electrode localization (b) and relative contribution of each contact to the normalized node strength of the surrogate corrected mutual information matrix M (c) for patient I-2 (first seizure). The spheres in panel c are centered at the positions of the intracranial electrode contacts. Their volume is proportional to the peri-ictal channel-wise mean of the node strength. The implantation scheme in this patient was fully symmetric. For simplicity, only electrodes in the left hemisphere are shown in panel c. The color code is as follows: red, channels included in the RBT; blue, channels belonging to the SOZ; magenta, overlap OVL, i.e. channels that were resected and belonged to the SOZ; black, channels NON that neither belonged to the RBT nor to the SOZ. Channel labels are: TPL, temporo-polar left; TBL, temporo-basal left; TLL, temporo-lateral left; DEL, depth electrode left. A movie showing the contribution of all four measures on the implantation scheme of the left hemisphere in 3D is available in the supplementary material (S1 Movie).

**Fig 2:**
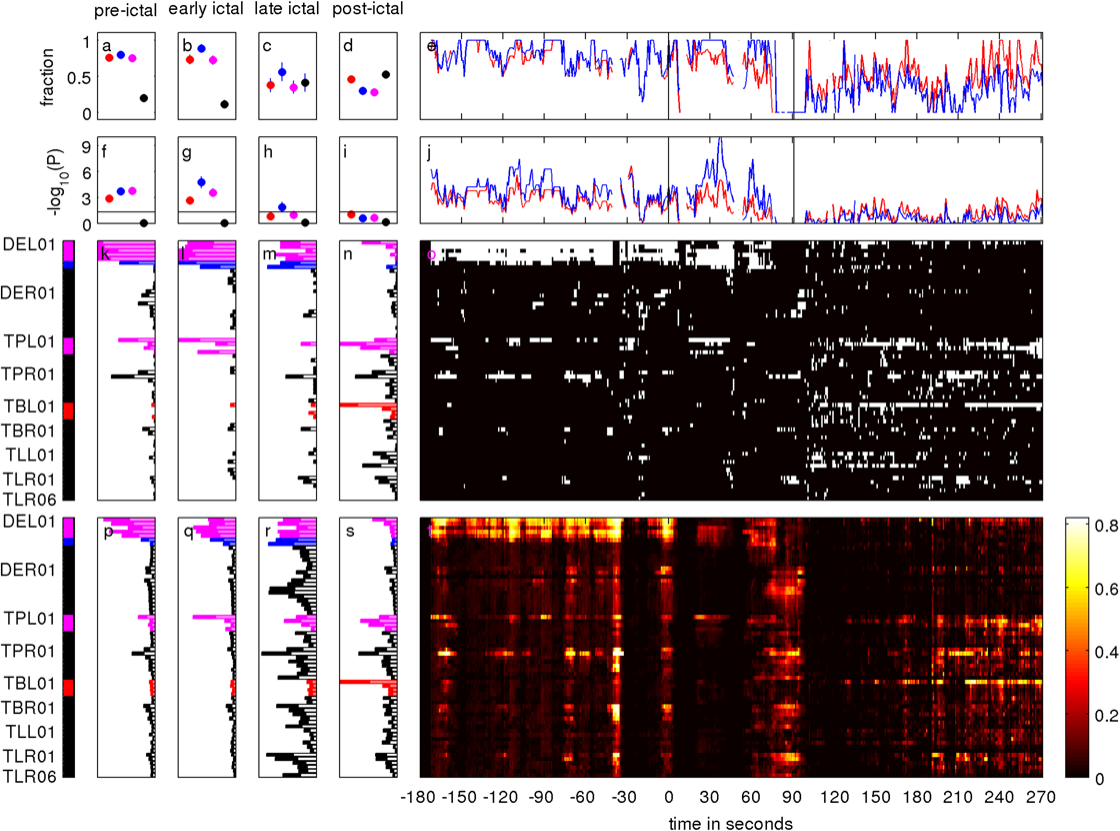
Peri-ictal evolution of quantifiers derived from the normalized node strength of the surrogate corrected mutual information matrix M of iEEG signals. The seizure starts at time point zero. Visually determined seizure onset and termination are indicated by vertical lines in the panels e and j. iEEG channels recording from RBT are indicated in red, the SOZ in blue and the overlap of both in magenta on the very left. Channels belonging to none of these zones are indicated in black. The color-scale figure in panel t shows the temporal evolution of each iEEG channel's normalized node strength of M. The vertical bar plots in panels p, q, r and s display the mean channel contribution in each of the following phases: pre-ictal, early ictal, late ictal and post-ictal. All these bar plots are scaled for optimal display. The bars are light shaded, whereas the standard error of the mean contribution is displayed in full color. The temporal evolution of salient (white) and normal channels (black) is shown in panel o. The vertical bar plots in panels k, l, m and n show the channel-wise mean prevalence of salient values in the four peri-ictal phases. For better comparison, here, all bar plots are shown in the same range 0 to 1.1. Panel e shows the temporal evolution of the fraction of salient values falling into the RBT (red) and the SOZ (blue). Panels a, b, c and d show the means over all four zones Z = {RBT,SOZ,OVL,NON} during the four peri-ictal phases. The 95% confidence interval for the mean is displayed as whiskers. The negative logarithm of the probability for randomly finding the observed or a larger amount of salient channels in the RBT (red), the SOZ (blue) and the overlap OVL of both (magenta) is shown in panel j as a function of time and in panels f, g, h and i as mean over the four peri-ictal phases. The 95% confidence interval for the mean is displayed as whiskers and the horizontal line indicates an approximation to the significance threshold -log_10_(0.05) = 1.30 for temporal means.

**Fig 3:**
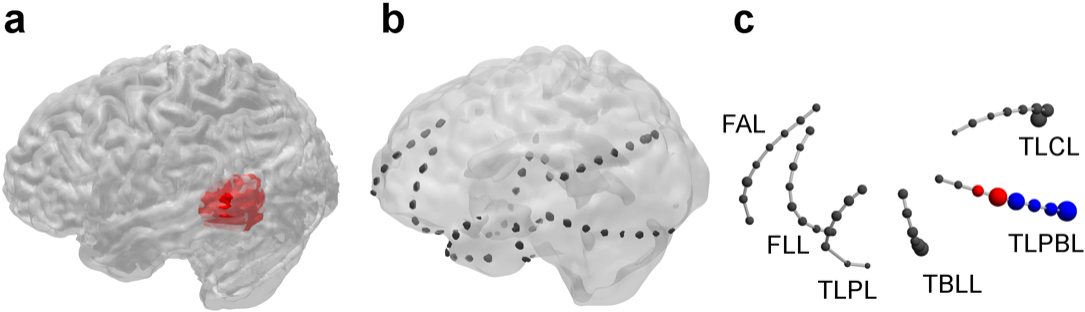
Same neuroanatomical representation as in Fig 1 but for the first seizure of patient IV-1. The color code is as follows: red, channels included in the RBT; blue, channels belonging to the SOZ; black, channels NON that neither belonged to the RBT nor to the SOZ. In contrast to patient I-2 in Fig 1, there was no overlap OVL between SOZ and RBT. Channel labels are: FAL, frontal anterior left; FLL, frontal lateral left; TLPL, temporo-lateral to polar left; TBLL, temporo-basal left; TLPBL, temporo-lateral to parieto-basal left; TLCL, temporo-lateral to cranial left. A movie showing the contribution of all four measures on the implantation scheme in 3D is available in the supplementary material (S2 Movie).

**Fig 4:**
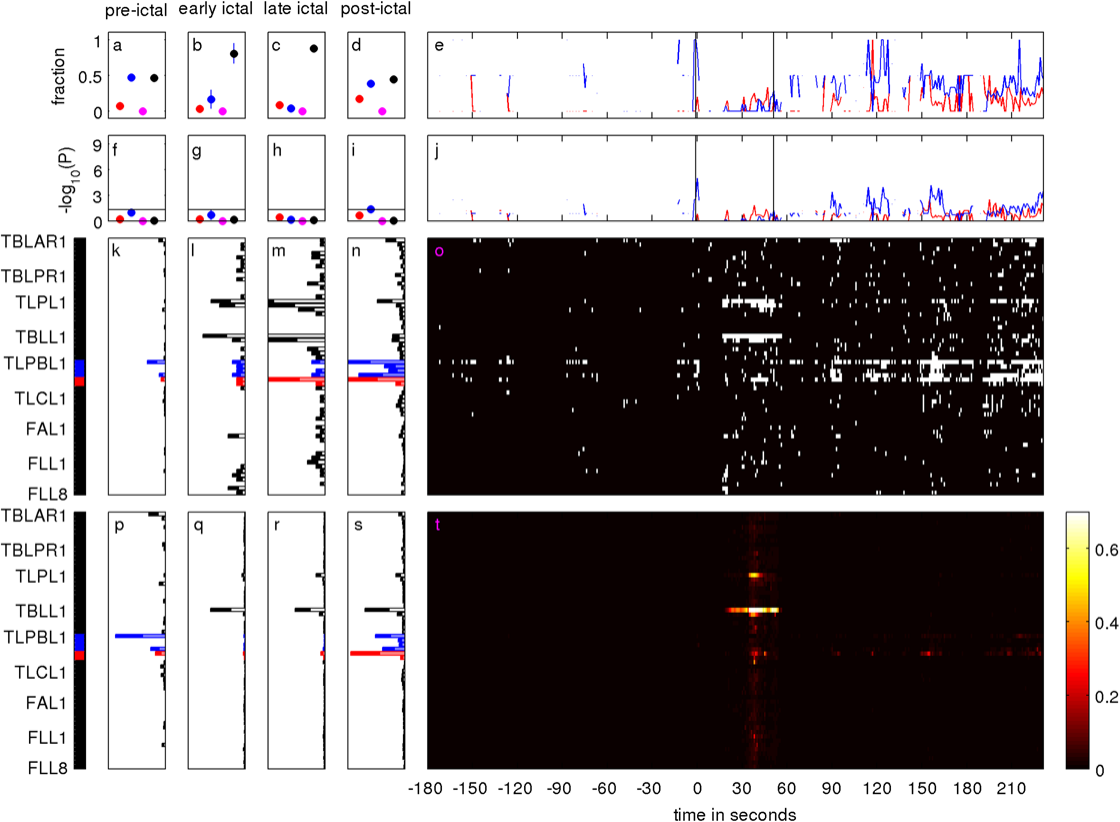
Same as Fig 2 but for the first seizure of patient IV-2. For time steps where no salient channels were identified, the quantifiers $F_{Z}^{M}$ and $L_{Z}^{M}$ are undefined, leading to discontinuities of their respective time courses (panels e and j).

We illustrate the temporal evolution of the qEEG measures using as an example the surrogate corrected mutual information matrix M for the first seizure of patients I-2 and IV-1 in Figs 1 to 4. Results for all four measures and both included seizures of these two patients are compiled in S1–S4 Figs. These figures clearly demonstrate that the peri-ictal dynamics of most qEEG measures is highly stereotypical, i.e. it is very similar for different seizures of the same type in an individual patient.

Patient I-2 remained seizure free after surgery during the follow-up period of three years. Fig 1 shows the anatomical relationship of the RBT and intracranial contacts (panels a and b), as well as the peri-ictal mean contribution of each electrode to the qEEG measure M (panel c). All n = 64 iEEG channels were free of permanent artifacts and therefore were all included in the analysis. The overlap OVL between the SOZ (11 channels) and the RBT (13 channels) was 9 channels (set union n-NON = 15 channels, Jaccard index J = OVL/(n-NON) = 0.6). The peri-ictal evolution of the normalized node strength of the surrogate corrected mutual information matrix M during the first seizure recorded is displayed in panel t of Fig 2. Ahead of the seizure, channels DEL01 to DEL05 of the left mid-temporal depth electrode revealed the largest node strength, followed by the left temporo-polar channel TPL01 and the right temporo-polar channel TPR02. The channels DEL01 to DEL05 and TPL01 were all in the overlap of the SOZ and the RBT, whereas channel TPR02 was recorded from brain tissue that was not resected. During the first 2/3 of the seizure the average normalized node strength of M decreased, before a re-increase occurred during the last 1/3 of the seizure. Interestingly, the node strength pattern before seizure termination was much more uniformly distributed than the one observed at earlier times. After seizure termination the strongest contributions to the normalized node strength of M were due to different channels than before the seizure: TPL01 to TPL04 (all in the overlap of SOZ and RBT) and the temporo-basal channel TBL01, which was in the RBT but not in the SOZ. Bar plots of temporal means over the pre-ictal, early ictal (first half of the seizure), late ictal (second half of the seizure) and post-ictal phase are shown in panels p, q, r and s, respectively.

The temporal evolution of saliency in the normalized node strength of the mutual information matrix M is shown in binarized form in panel o (white: salient channel, black: non-salient channel) and bar plots of temporal means over the four phases are given in panels k, l, m and n. In the pre-ictal as well as in the early and late ictal phases the saliency pattern was very stable over time, with largest frequency of salient values in the overlap OVL of RBT and SOZ (channels DEL01 to DEL06 and TPL01 to TPL04). Ahead of seizure termination the amount of salient channels decreased and concentrated on channels recording from the right hemisphere (contralateral to the visually defined SOZ) which were consistently neither in the SOZ nor in the RBT. After seizure termination, the main prevalence of salient values was in channels TPL01 to TPL04 and TBL01. All of them were in the RBT and most of them in the SOZ. Nevertheless, the post-ictal strength pattern was substantially different from the pre-ictal and early ictal pattern.

The fraction of salient channels of the node strength of M falling into each of the zones RBT and SOZ varied between 0.5 and 1 pre-ictally (panel e). None of the fractions in the RBT, the SOZ or in OVL was distinguished, whereas hardly any salient channel fell into NON (panel a). The configuration changed substantially during the seizure: The fraction of salient values $F_{Z}^{M}$ in the three zones RBT, SOZ and OVL decreased during seizure evolution and $F_{\text{SOZ}}^{M}$ became larger than $F_{\text{RBT}}^{M}$ and $F_{\text{OVL}}^{M}$. In contrast, the fraction of salient values $F_{\text{NON}}^{M}$ in the channels belonging neither to the RBT nor to the SOZ increased (panels b and c). After seizure termination the fraction of salient values in the zones RBT, SOZ and OVL was unstable, but on average remained smaller than in the pre-ictal and early ictal phase. Now, $F_{Z}^{M}$ had a similar value in the zones NON and RBT and was larger than in the zones SOZ and OVL (panel d).

The temporal evolution of the log probability $L_{Z}^{M}$ is displayed in panel j. Ahead of the seizure this value varied for between 2 and 6 for zones Z = {RBT,SOZ}, equivalent to p-values in the range between 0.01 and 10^−6^ and thus indicating a non-random association. Also on average $L_{Z}^{M}$ was larger than -log_10_(0.05) = 1.30 (panel f), a value that can heuristically be seen as the significance threshold for temporal averages. None of the zones Z = {RBT,SOZ,OVL} had a clearly larger value than any other. During seizure $L_{Z}^{M}$ increased up to the value 9 for the SOZ (overlap significance P = 10^−9^). After the seizure $L_{Z}^{M}$ dropped and varied around the heuristic significance threshold. The largest post-ictal values were obtained for $L_{\text{RBT}}^{M}$.

In contrast to patient I-2 surgery outcome was unfavorable in patient IV-1 (Fig 3). Out of 62 implanted iEEG contacts, n = 59 channels recorded signals free of permanent artifacts. As the visually defined SOZ overlapped with the functional language area, it was decided to restrict palliative resection to the vicinity of the SOZ to minimize the risk of post-operative neurological deficits. In consequence, the overlap OVL between the visually defined SOZ (4 channels) and the RBT (2 channels) was empty. Retrospective qEEG analysis showed that in patient IV-1 large normalized node strength of the surrogate corrected mutual information matrix M were much more confined in space and time (panel t of Fig 4) than for patient I-2. Before and after the seizure salient values were mainly located in the left temporo-latero-posterio-basal iEEG channels TLPBL1 to TLPBL6, some of which were in the RBT and some of which in the SOZ (panels k and n). During the seizure, salient values were also visible on channels of the left temporo-latero-polar (TLPL) and left temporo-basal-lateral (TBLL) strip electrodes (panels l and m), which neither corresponded to the SOZ nor to the RBT. Throughout the peri-ictal recording the fraction $F_{\text{RBT}}^{M}$ of salient channels in the RBT was much lower than in patient I-2. Before and after seizure $F_{\text{SOZ}}^{M}$ and $F_{\text{NON}}^{M}$ were close to 0.5 (panels a and d). $L_{Z}^{M}$ was smaller than in patient I-2 for any of the three zones of interest Z = {RBT,SOZ,OVL}. Only in the pre- and post-ictal phase the overlap with the SOZ came close to statistical significance (panel f).

### Summary statistics for qEEG measures

**Fig 5:**
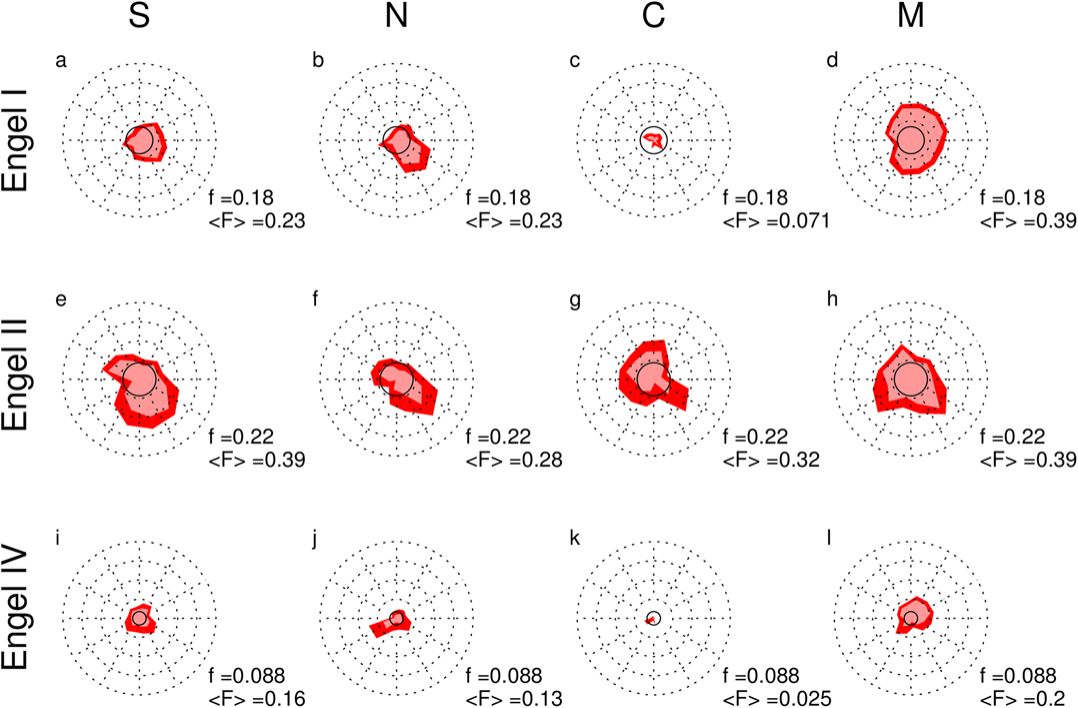
Peri-ictal radar plots of the fraction of salient values of measure X = {S,N,C,M} in the radiologically confirmed RBT for all 38 seizures in all 16 patients. Each row shows the data of one outcome subgroup (Engel class I: 6 patients, 14 seizures; class II: 5 patients, 10 seizures; class IV: 5 patients, 14 seizures), across the four different measures (S, absolute EEG slope; N, number of forbidden ordinal patterns; C, surrogate corrected cross-correlation; M, surrogate corrected mutual information). The data is arranged clockwise with the upper right quarter corresponding to three consecutive epochs of one minute duration immediately before seizure onset. The lower half corresponds to the scaled seizure time between seizure onset and termination, and the upper left quarter corresponds to three consecutive one-minute epochs immediately after seizure termination. The red polygons illustrate the temporal profile of the mean $F_{\text{RBT}}^{X}$ and the parametrically estimated 95% confidence interval of the mean is displayed by the thickness of the polygon outline. Broken circular lines correspond to fractions 0.25, 0.5, 0.75 and 1.0 from inside to outside. The continuous circular line corresponds to the fraction f of salient channels expected from the size of the RBT. The mean <F> as estimated from the circle area is given in the lower right corner of each plot. Note that the means given here can deviate from the medians used in the text. Analog figures for the visually defined seizure onset zone (SOZ), its overlap OVL with the RBT and channels contributing to none of these zones NON are given in S5, S6 and S7 Figs.

**Fig 6:**
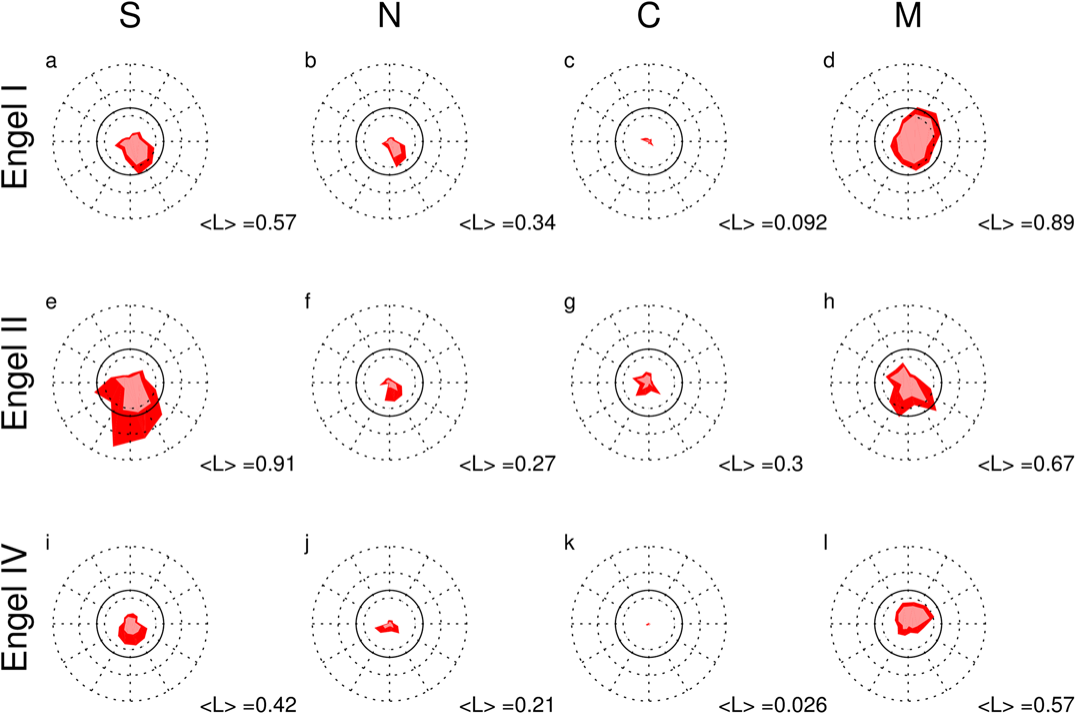
Peri-ictal radar plots of the mean $L_{\text{RBT}}^{X}$ for all 38 seizures in all 16 patients. Note that segment-wise mean log probabilities are displayed, not logarithms of segment-wise p-values. The figure elements are analog to Fig 5 with the exception that dashed circular lines heuristically correspond to p-values of 0.1, 0.01 and 0.001 from inside to outside. The continuous circular line corresponds to p = 0.05. The mean <L> as estimated from the circle area is given in the lower right corner of each plot. Note that the means given here can deviate from the medians used in the text. Analog figures for the visually defined seizure onset zone (SOZ), its overlap OVL with the RBT and the channels contributing to none of these zones NON are given in S8, S9 and S10 Figs.

In Figs 5 and 6 we illustrate the peri-ictal evolution of our qEEG quantifiers $F_{\text{RBT}}^{X}$ and $L_{\text{RBT}}^{X}$ for all 38 seizures of all 16 patients. These radar plots enable an intuitive clockwise visualization of the temporal evolution before (upper right quadrant), during (lower half) and after seizures (upper left quadrant). Analog representations for the SOZ, its overlap OVL with the RBT and channels contributing to none of these zones NON are given in S5–S10 Figs.

The class-wise mean fraction $F_{\text{RBT}}^{X}$ of salient channels that were resected during epilepsy surgery is shown in Fig 5 together with the value f expected from the size of the RBT under uniform distribution (fully drawn circles). The class-averaged peri-ictal mean <F> (proportional to the square root of the area included in the polygons) was largest for X = M (last column), followed by S (first column), N (second column) and C (third column). Outcome class II showed the largest resected fraction of salient values for all qEEG measures (second row), followed by class I (first row) and Engel class IV (third row). Note that in patients of Engel class II also the absolute and relative size of the RBT was largest though the difference lacked significance (Table 2). For the unfavorable outcome class IV the average resected fraction of salient values was hardly ever larger than 25% for any qEEG measure in the peri-ictal evolution. In contrast, for the favorable outcome classes I and II we found time periods where the class-wise mean $F_{\text{RBT}}^{X}$ was close to 50% despite the fact that the RBT comprised less than 25% of the channels on average. In patients who became completely seizure free (Engel class I) roughly half of the salient channels of the surrogate corrected mutual information matrix M found during the pre-ictal and early ictal phases were resected. The same was the case for channels showing salient values of the number of forbidden ordinal patterns N in the early ictal phase.

For the fraction of salient values in the SOZ and in the overlap OVL between SOZ and RBT we found similar results (S5 and S6 Figs) with the difference that large values of $F_{\text{SOZ}}^{X}$ were more peaked in the early ictal phase, especially for X = S. Channels that neither recorded from the RBT nor form the SOZ had a consistently smaller fraction of salient values than expected from the size of zone NON only for qEEG measure M in the full peri-ictal epoch and for S and N in the early ictal phase (S7 Fig).

With exception of the pre-ictal and early ictal phases of M and the early ictal phase of S in Engel classes I and II the class-averaged peri-ictal mean of $L_{\text{RBT}}^{X}$ (polygon areas in Fig 6) hardly ever during the peri-ictal evolution reached the heuristic border of significance (L>1.3 or P<0.05, fully drawn lines). In terms of qEEG measures it decreased in exactly the same sequence as $F_{\text{RBT}}^{X}$, i.e. M>S>N>C. The number of forbidden patterns N and the surrogate corrected cross-correlation matrix C (second and third column, respectively) had much smaller polygon areas, indicating on average smaller significance of the overlap between salient channels of these two measures and the RBT. In terms of outcome classes, the polygon areas decreased from Engel class II (second row) over I (first row) to IV (third row).

The association between salient and SOZ channels became significant in the early ictal phase for qEEG measure S and in the pre-ictal phase for M (S8 Fig). To lesser extent a similar observation was made for the overlap OVL between RBT and SOZ (S9 Fig). In contrast, no association was found between salient channels and those neither in the RBT nor in the SOZ (S10 Fig). For all Engel classes the salient channels of the absolute signal slope S were stronger associated with the SOZ in the early ictal phase than with the RBT (panels a, e and i of S8 Fig and Fig 6).

We estimated the peri-ictal means of the fraction of salient values $F_{Z}^{X}$ of measure X in zone Z and the corresponding log probability $L_{Z}^{X}$ seizure-wise from the area included in the radar plots. p-values of a Kruskal-Wallis test for class-wise different rank sum of $F_{Z}^{X}$ (Table 3) were significant only for the RBT and qEEG measures M, S and N. For all these measures post-hoc testing for pair-wise differences showed that the medians in Engel classes I and II were significantly larger than in class IV, whereas there was no significant difference between classes I and II (S: 25.8%, 34.6%, 19.9%; N: 27.0%, 31.5%, 11.1%; M: 29.1%, 32.7%, 13.9% for classes I, II and IV, respectively). In contrast, for the SOZ and the overlap OVL significance was never reached.

**Table 3:** Testing associations between the Engel class and the seizure-wise mean fraction of salient values $F_{Z}^{X}$ in the regions of interest.

	RBT		SOZ		OVL		NON	
	1^st^	2^nd^	1^st^	2^nd^	1^st^	2^nd^	1^st^	2^nd^
**S**	**0,041**	**II>IV (p = 0.022)**	0,182	n.a.	0,343	n.a.	0,096	n.a.
**N**	**0,031**	**I>IV (p = 0.048), II>IV (p = 0.016)**	0,65	n.a.	0,277	n.a.	0,263	n.a.
**C**	0,237	n.a.	0,754	n.a.	0,163	n.a.	0,075	n.a.
**M**	**0,013**	**I>IV (p = 0.012), II>IV (p = 0.014)**	0,843	n.a.	0,142	n.a.	0,825	n.a.

If a 1^st^ level Kruskal-Wallis test rejected the null hypothesis that all data stem from the same distribution, pair-wise 2^nd^ level Mann-Whitney-Wilcoxon tests were used to assess differences between the class-wise medians. Significant results are highlighted in boldface (uncorrected p-values).

Table 4 shows the test results for class differences of $L_{Z}^{X}$. Here significant class differences were found for measures M and N in zone NON. For measure M the Engel class II had significantly larger median log probability than classes I and IV (0.096, 0.174, 0.061) and Engel classes I and II had significantly larger median log probability in measure N than class IV (0.110, 0.092, 0.064). Note that none of the log probabilities leading to class differences came even close to the heuristic significance threshold L>1.3 for means.

**Table 4:** Testing associations between the Engel class and the log probability $L_{Z}^{X}$ in the regions of interest.

	RBT		SOZ		OVL		NON	
	1^st^	2^nd^	1^st^	2^nd^	1^st^	2^nd^	1^st^	2^nd^
**S**	0,442	n.a.	0,478	n.a.	0,497	n.a.	0,666	n.a.
**N**	0,377	n.a.	0,534	n.a.	0,3	n.a.	**0,049**	**I>IV (p = 0.048), II>IV (p = 0.026)**
**C**	0,202	n.a.	0,672	n.a.	0,132	n.a.	0,068	n.a.
**M**	0,213	n.a.	0,538	n.a.	0,239	n.a.	**<0.001**	**II>I (p = 0.006), II>IV (p<0.001)**

Statistics and result representation is as in Table 3.

## Summary and Discussion

We have analyzed the spatial relation between iEEG channels with focally salient values in four qEEG measures and the resected brain tissue (RBT) as well as the seizure onset zone (SOZ). The exact localization of the RBT and of the electrodes recording the iEEG signals was confirmed radiologically by coregistration of pre- and post-surgical MRI and CT imaging. In contrast to this quantitative approach, the SOZ as well as seizure onset and termination times were defined by the current clinical gold standard, i.e. visual EEG reading by experienced epileptologists. The qEEG measures were selected to be representative for four different classes of signal analysis methods: linear univariate (S), nonlinear univariate (N), linear multivariate (C) and nonlinear multivariate (M). The methodology was retrospectively applied to 38 peri-ictal iEEG epochs from 16 epilepsy patients (three patients had two seizure types) with favorable (6 patients in Engel class I and 5 patients in class II) and unfavorable (5 patients in Engel class IV) post-surgical seizure control.

### Main findings

Our main findings are the following. First, regardless of the used qEEG measure, the median fraction of salient channels in the RBT was larger in the favorable outcome classes Engel I and II than in the unfavorable outcome class IV, where on average less than 20% of the channels showing salient values were resected (Fig 5). For X = {M,S,N} the fraction of salient values in the RBT was significantly larger in the favorable than in the unfavorable outcome classes, whereas in the SOZ or in the overlap OVL we did not find a class difference. Second, in all outcome classes the average fraction of salient values in the RBT was larger for the absolute signal slope S and the normalized node strength of the surrogate corrected mutual information matrix M than for the number of forbidden ordinal patterns N and the node strength of the surrogate corrected cross-correlation matrix C (Fig 5). Third, the association between iEEG channels defined as salient and three out of four of the studied zones Z = {RBT,SOZ,OVL} intermittently reached beyond than chance level only for the absolute signal slope S and the node strength of matrix M and only for the favorable outcome classes I and II (Fig 6). Association between salient iEEG channels and the zone NON was never significant.

Despite larger median values in favorable than in unfavorable outcome groups, $L_{\text{RBT}}^{X}$ and $F_{\text{RBT}}^{X}$ was often larger in Engel class II than I. The 95% confidence intervals of the means were also wider in class II. This rather counter-intuitive observation may be explained by two reasons. First, our patients with outcome class II could form a less homogeneous group than those of classes I and IV. Second and more likely, the fraction of iEEG channels in the overlap between SOZ and RBT was largest in class II (median fraction of 3.4%, 3.9% and 0.8% in Engel classes I, II and IV, respectively). This might have introduced a bias towards larger $L_{Z}^{X}$ and $F_{Z}^{X}$ in this subgroup. Note that despite different channel fraction in the overlap we did not observe a significant class difference in the Jaccard index for overlap between RBT and SOZ (Table 2). The reason is that also the union of channels recording from the RBT and the SOZ represented a larger channel fraction in outcome class II than in IV. We interpret this finding in the sense that post-surgical seizure control tended to be better for patients where a larger fraction of iEEG channels recorded from the hypothetical EZ, i.e. for those patients with better pre-implantation hypotheses about the localization of the epileptogenic brain areas.

Apart from these trends we did not find any statistical differences between the clinical parameters of the three outcome groups. We interpret this as corroborating evidence that the above summarized results are not an artifact of patient selection but underline the value of quantitative analysis of peri-ictal iEEG recordings added to classical expert visual inspection.

## Discussion

For our study we selected qEEG measures that represent four classes of signal analysis algorithms. It is plausible that alternative ways of assessing similar signal properties would yield similar results. The absolute signal slope S [35] is a simple univariate and linear, but of course not the only qEEG measure that has been designed to detect epileptiform signals by its high frequency content. As alternatives the “joint sign periodogram event characterization transform algorithm” (JSPECT) [66], the “epilepticity index” [16] or high frequency oscillations [18] are conceivable. Alternatives to the univariate but nonlinear number of forbidden ordinal patterns N [41] for quantification of signal determinism could be the amplitude-based “nonlinear prediction error” [67,68] or its rank-based extension, the “nonlinear prediction score” [69,54]. Related are attempts to determine approximations to the correlation integral from EEG time series [70,71]. Also for quantification of linear or nonlinear signal interrelation there are many alternatives to the chosen surrogate corrected measures C and M of [46], see [9,10] for reviews. Without completeness we mention linear cross-coherence [72], the nonlinear correlation coefficient [73,74], phase synchronization [75], Granger causality [76] and cross-predictabilities [77].

Applying qEEG measures and identifying the RBT radiologically we centered our study around quantifiable information. We tried to avoid any potential ambiguity or inter-rater variability as might occur during visual EEG or MRI interpretation. The proposed method might be especially important in the subgroup of patients, where the current clinical gold standards, i.e. EEG “reading” by trained experts and visually defining the SOZ remain ambiguous. The safety of high-field MRI with implanted electrodes is still a matter of debate, thus co-registration of pre- and post-surgical MRI with post-implantation CT is currently the method of choice to define the precise position of intracranial electrodes and the RBT.

Our finding that the differentiation between favorable and unfavorable outcome was possible for $F_{\text{RBT}}^{X}$ but not for $F_{\text{SOZ}}^{X}$ is consistent with growing evidence that widespread networks might be more relevant for seizure generation, evolution and termination than a single brain region (i.e. the “focus”) where seizures actually start from [78,22]. In a recent study [79] it was found that despite larger average extent of SOZ resection in pediatric patients with favorable outcome (2/3 resected as compared to 1/3 resected in unfavorable outcomes), complete resection of the SOZ was required only in one out of eight cases to achieve seizure freedom.

The dynamic and data-driven definition of salient qEEG values has the advantage that these prominent values can be analyzed whenever they occur. Specifically, this implies that no assumption has to be made when focal saliency might be most prevalent (e.g. in the early ictal phase, where “early” has to be specified somehow). This concept has the additional advantage that inter-ictal or sub-clinical events that might escape the observer's attention can be detected objectively and can be used for analysis.

Reporting $F_{Z}^{X}$ and $L_{Z}^{X}$ is better suited to situations without a “ground truth” than the use of classification accuracies like sensitivity and specificity, positive and negative predictive values, or likelihood and odds ratios [80]. In epilepsy surgery there is not yet a method to simulate different surgical procedures in order to evaluate if they are effective. Moreover, in successful cases the actual RBT is most probably larger than minimally needed (i.e. the epileptogenic zone EZ), which negatively biases most accuracy quantifiers. The fraction of salient values, however, has the property to saturate at 100% even if the RBT is larger than the EZ.

## Limitations and Outlook

Our study has several limitations. First, the number of patients included into the study is limited (38 seizures from 16 patients), retrospective in nature, derived from a single center epilepsy surgery program and heterogeneous in terms of etiology and electrode implantation scheme. Although we were able to show that our results are robust against a number of confounding demographic and disease-related variables, it is unclear at present how our results will generalize to larger or prospective cohorts.

Second, the chosen scheme for detection of salient values is suitable for skewed distributions, but it is still a parametric concept. A different choice of the whisker parameter w produces a different number of salient values. We used the standard choice w = 1.5 and did not investigate the influence of different values of w on our results. As a further technical aspect, the IAAFT surrogate generation is computationally expensive, especially in the multivariate case where phase randomization is performed under the constraint of conserved phase relationships between all iEEG channels. For larger studies or prospective application of the surrogate corrected mutual information matrix M it has to be investigated whether faster algorithms, e.g. constrained phase randomization without amplitude adaption or the iterative procedure [81], are sufficient for our purposes.

In this study we applied the Engel seizure outcome classification, being aware of its limitations due to the dependency on the patient's and his/her family's perception and long-term reports of seizure rate, which must be considered with caution [82].

Finally, one inherent difficulty in this type of study–also mentioned above–is that even the RBT, as delineated by MRI, may be considered as an imperfect benchmark, since we cannot exclude that any other surgical approach might have led to a similar post-surgical outcome in a given patient, i.e. in a situation where the EZ is unique. In our opinion it will be hard to address or even resolve this issue satisfactorily. From a more pragmatic point of view, the RBT can easily and objectively be assessed with current imaging methods.

The clinical interpretation of some qEEG measures and results may be complex despite of their objective mathematical definition. Whereas the absolute signal slope S has a straightforward interpretation in terms of high frequency or large amplitude signals, the interpretation of the number of forbidden ordinal patterns N or of the surrogate corrected measures C and M is less obvious. Bringing qEEG closer to clinical understanding will be a task for future investigations.

At present we cannot prove that resection of brain tissue generating salient values in certain qEEG measures automatically leads to post-surgical seizure control. Rather, we believe that our findings suggest that special attention should be given to saliency-generating iEEG channels during pre-surgical evaluation and surgery planning. Primary candidates are salient channels of the absolute signal slope S in the early ictal phase or of the normalized node strength of the surrogate corrected mutual information matrix M in immediate pre-ictal epochs (Figs 5 and 6). Occasionally, it might be possible to adapt the intended resection target to increase the fraction of salient values in the anticipated RBT accordingly. The proposed methods may be further investigated in epilepsy surgery to estimate the expected outcome by simulation of the anticipated RBT. In situations where different surgical approaches are under debate, the alternatives could be modeled and could contribute to the decision making on the best strategy to render the patient seizure free.

## Supporting Information

S1 Fig Compilation of peri-ictal results for all four qEEG measures during the first seizure of patient I-2. A: absolute signal slope S, B: number of forbidden ordinal patterns N, C: surrogate corrected cross-correlation matrix C, D: surrogate corrected mutual information matrix M (same data as Fig 2 of the main text). The arrangement of the panels A to D is identical to Figs 2 and 4 of the main text.
(TIFF)

S2 Fig Compilation of peri-ictal results for all four qEEG measures during the second seizure of patient I-2. The figure arrangement is identical to S1 Fig. Panel D is identical to Fig 4 of the main text.
(TIFF)

S3 Fig Compilation of peri-ictal results for all four qEEG measures during the first seizure of patient IV-1. The figure arrangement is identical to S1 Fig.
(TIFF)

S4 Fig Compilation of peri-ictal results for all four qEEG measures during the second seizure of patient IV-1. The figure arrangement is identical to S1 Fig.
(TIFF)

S5 FigPeri-ictal radar plots of the fraction $F_{\text{SOZ}}^{X}$ of salient values within the seizure onset zone (SOZ) for all 38 seizures in all 16 patients.The figure arrangement is analog to Fig 5 of the main text.(TIFF)

S6 FigPeri-ictal radar plots of the fraction $F_{\text{OVL}}^{X}$ of salient values within the overlap OVL of the RBT and the SOZ for all 38 seizures in all 16 patients.The figure arrangement is analog to Fig 5 of the main text.(TIFF)

S7 FigPeri-ictal radar plots of the fraction $F_{\text{NON}}^{X}$ of salient values within the channels contributing neither to the RBT nor to the SOZ (zone NON) for all 38 seizures in all 16 patients.The figure arrangement is analog to Fig 5 of the main text.(TIFF)

S8 FigPeri-ictal radar plots of the log probabilities $L_{\text{SOZ}}^{X}$ (seizure onset zone) for all 38 seizures in all 16 patients.The figure arrangement is analog to Fig 6 of the main text.(TIFF)

S9 FigPeri-ictal radar plots of the log probabilities $L_{\text{OVL}}^{X}$ (overlap of RBT and SOZ) for all 38 seizures in all 16 patients. The figure arrangement is analog to Fig 6 of the main text.(TIFF)

S10 FigPeri-ictal radar plots of the log probabilities $L_{\text{NON}}^{X}$ (channels contributing neither to the RBT nor to the SOZ) for all 38 seizures in all 16 patients.The figure arrangement is analog to Fig 6 of the main text.(TIFF)

S1 Movie 3D animation of the spatial layout of the intracranial electrodes implanted into the left hemisphere of patient I-2. Each sphere represents the position of one intracranial electrode. The sphere color corresponds to the analyzed zone, i.e. red, resected brain volume (RBT); blue, visually determined seizure onset zone (SOZ); magenta, overlap (OVL); black, neither of the above (NON). The sphere radius is proportional to the peri-ictal channel-wise mean of each qEEG measure during the first seizure (see main text for details). The layout is as in S1–S4 Figs: upper row, absolute signal slope S (left), number of forbidden ordinal patterns N (right); lower row, surrogate corrected cross-correlation matrix C (left), surrogate corrected mutual information matrix M (right).
(AVI)

S2 Movie Same as S1 Movie but for the first seizure of patient IV-1. (AVI)

## Abbreviations

CT: computed tomography
EEG: electroencephalography
EZ: epileptogenic zone
FLE: frontal lobe epilepsy
IAAFT: iterated amplitude adjusted Fourier transform surrogates
iEEG: intracranial EEG
IQR: inter-quartile range
LTLE: lateral temporal lobe epilepsy
MDEFT: modified driven equilibrium Fourier transform
MRI: magnetic resonance imaging
MTLE: mesial temporal lobe epilepsy
n.a.: not applicable
NON: channels neither in RBT nor in SOZ
OVL: overlap of RBT and SOZ
PLE: parietal lobe epilepsy
qEEG: quantitative EEG
RBT: resected brain tissue
SOZ: seizure onset zone
T1w: T1-weighted
