# Supplementary figures and images for: Resected Brain Tissue, Seizure Onset Zone and Quantitative EEG Measures: Towards Prediction of Post-Surgical Seizure Control
Source: PLoS One. 2015 Oct 29;10(10):e0141023. doi: 10.1371/journal.pone.0141023 (PMC4626164; doi:10.1371/journal.pone.0141023)

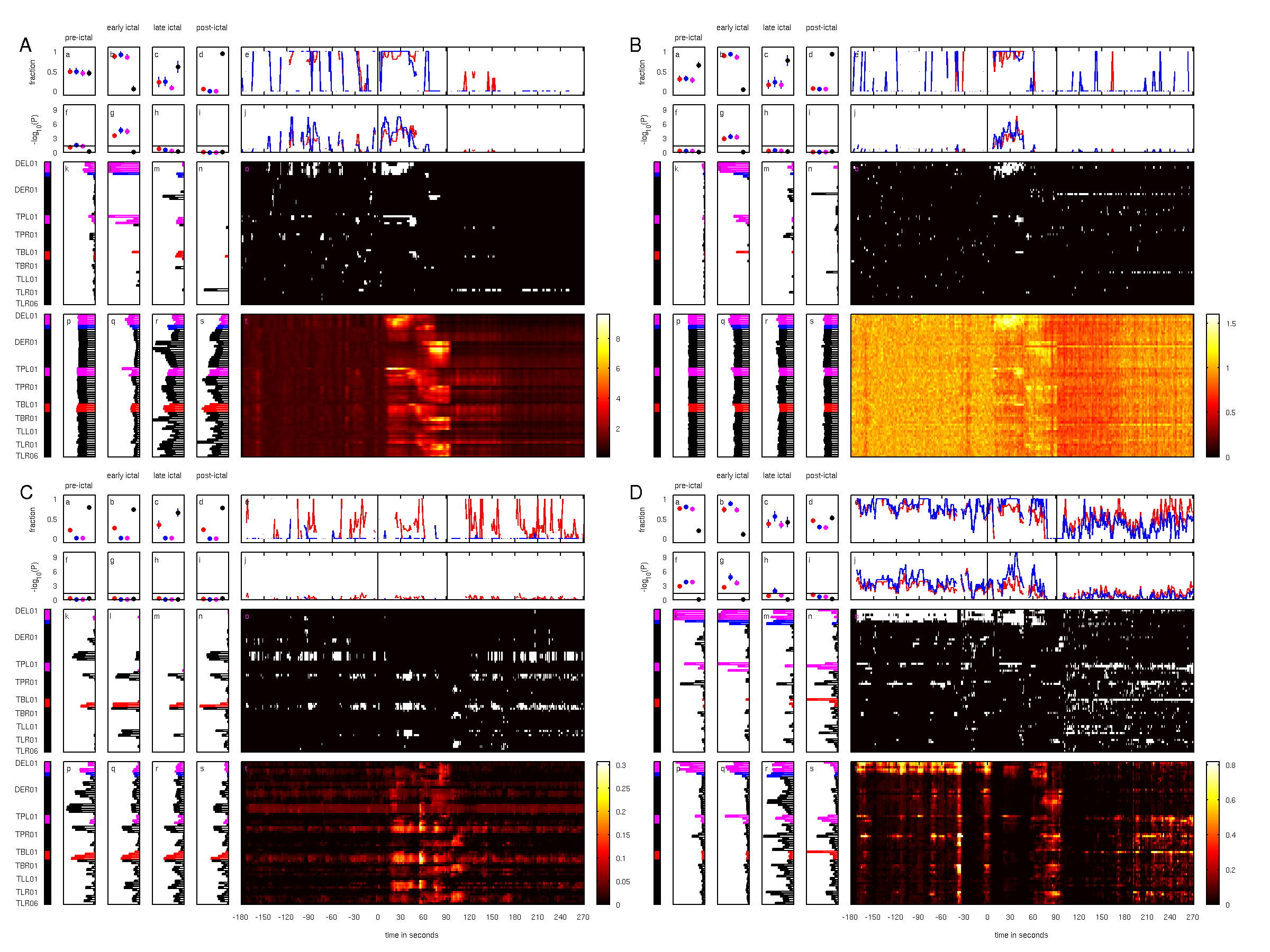

Supplement: S1 Fig — A: absolute signal slope S, B: number of forbidden ordinal patterns N, C: surrogate corrected cross-correlation matrix C, D: surrogate corrected mutual information matrix M (same data as Fig 2 of the main text). The arrangement of the panels A to D is identical to Figs 2 and 4 of the main text. (TIFF) [file pone.0141023.s001.tiff]

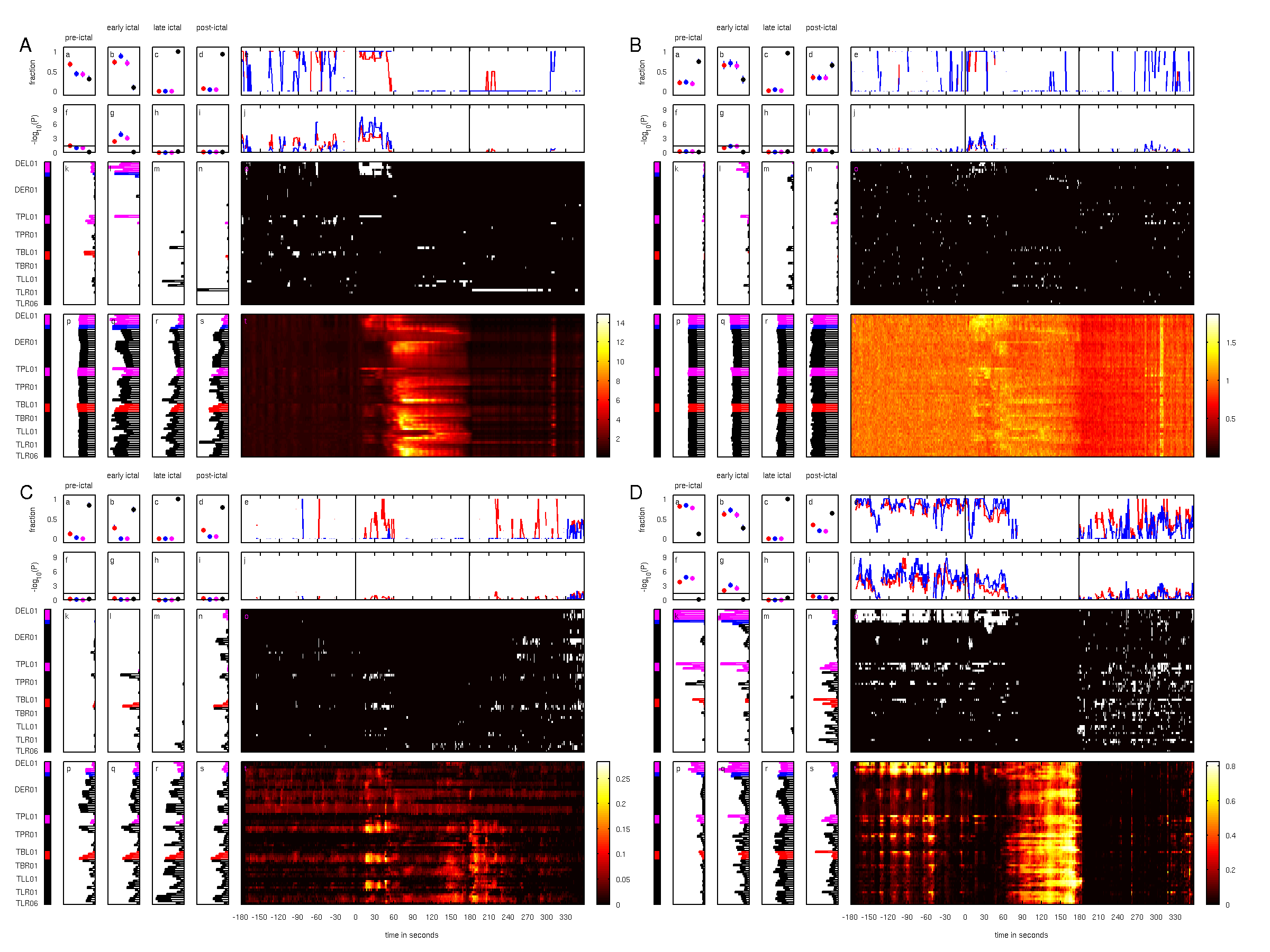

Supplement: S2 Fig — The figure arrangement is identical to S1 Fig. Panel D is identical to Fig 4 of the main text. (TIFF) [file pone.0141023.s002.tiff]

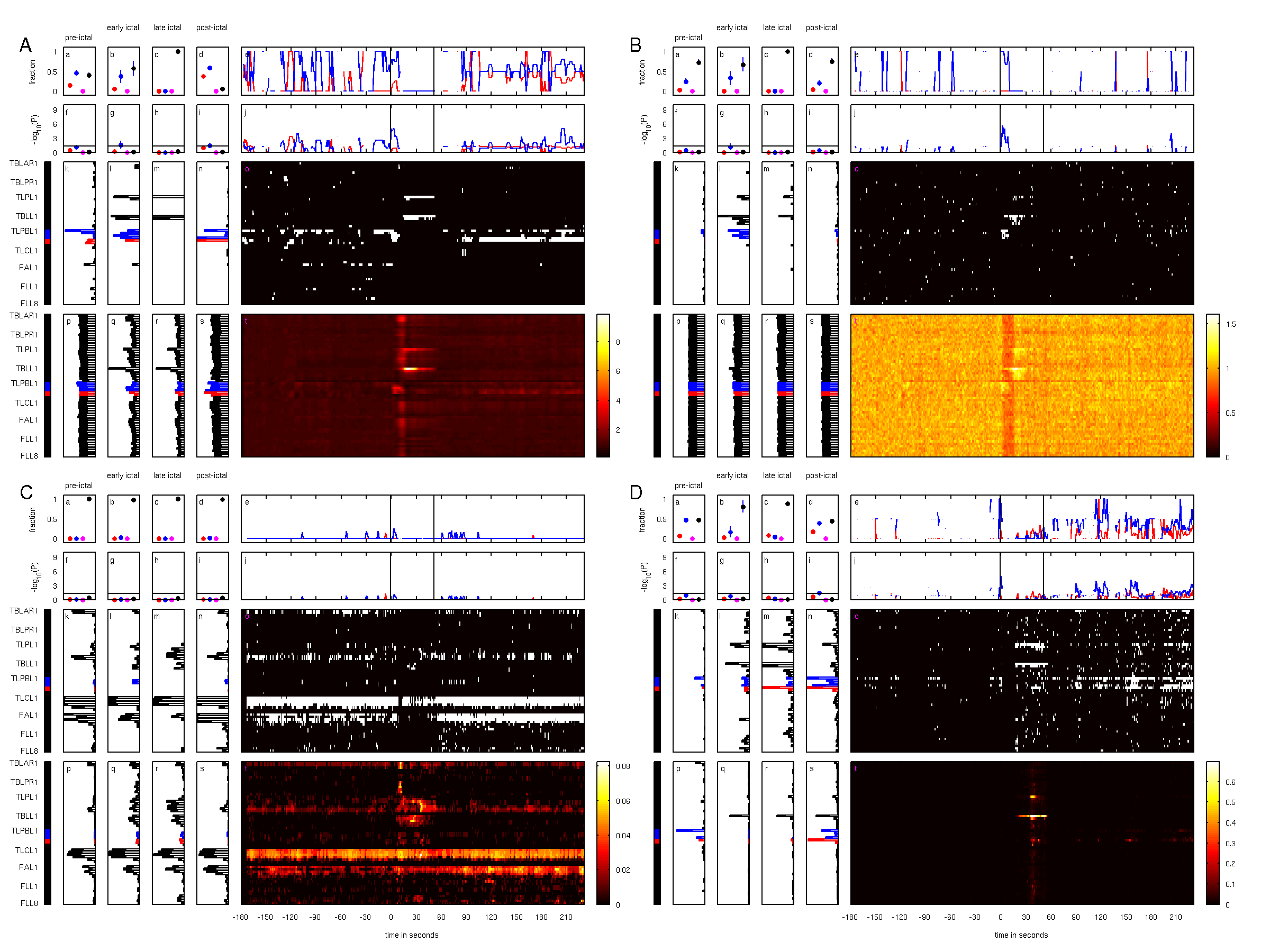

Supplement: S3 Fig — The figure arrangement is identical to S1 Fig. (TIFF) [file pone.0141023.s003.tiff]

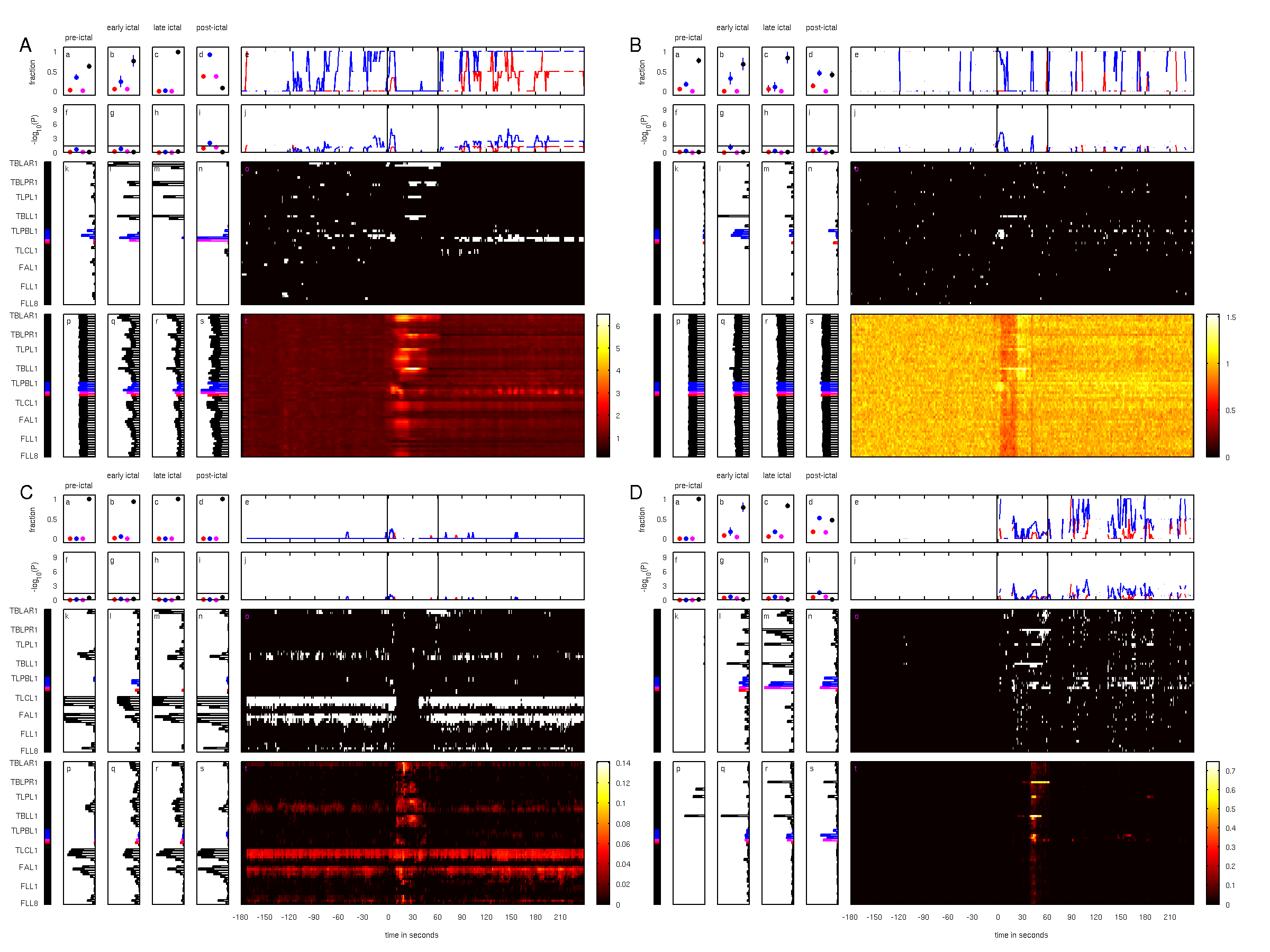

Supplement: S4 Fig — The figure arrangement is identical to S1 Fig. (TIFF) [file pone.0141023.s004.tiff]

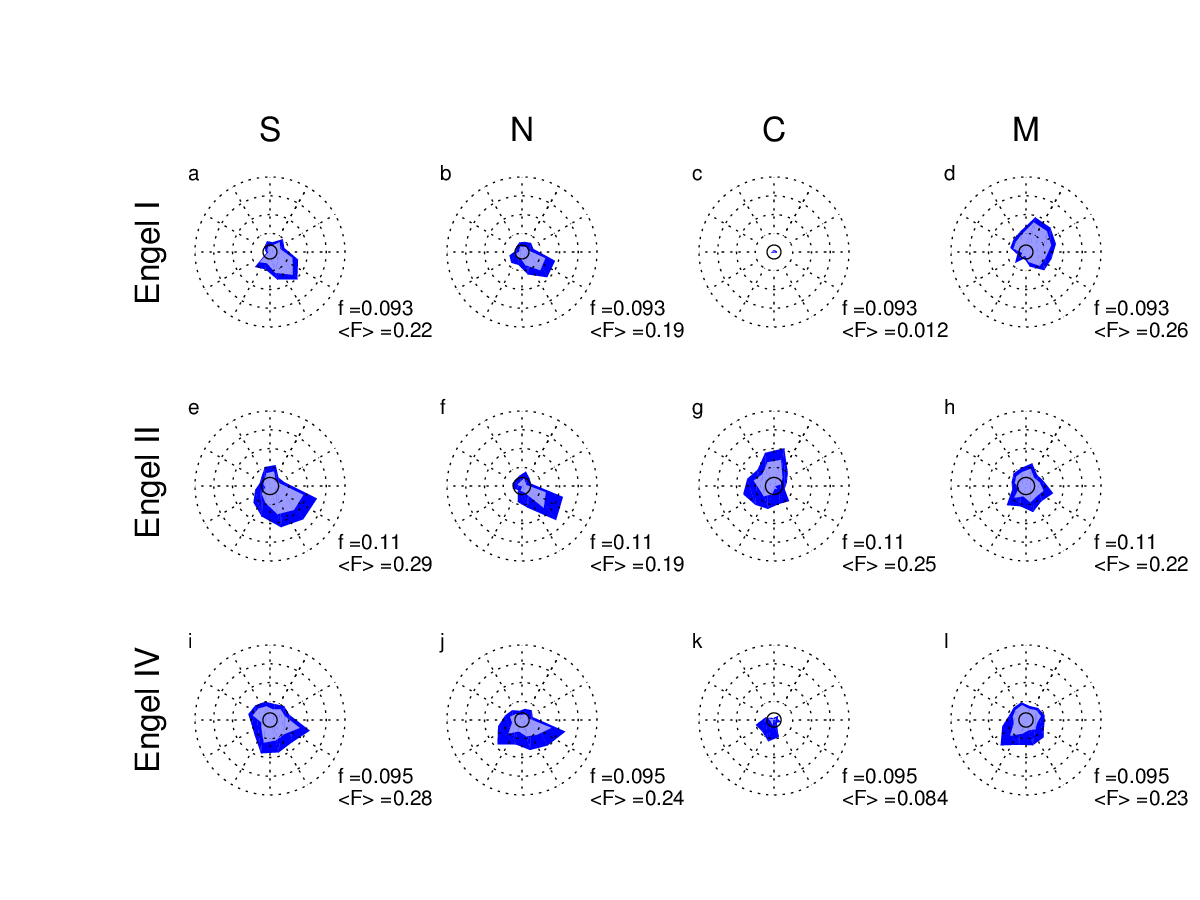

Supplement: S5 Fig — The figure arrangement is analog to Fig 5 of the main text. (TIFF) [file pone.0141023.s005.tiff]

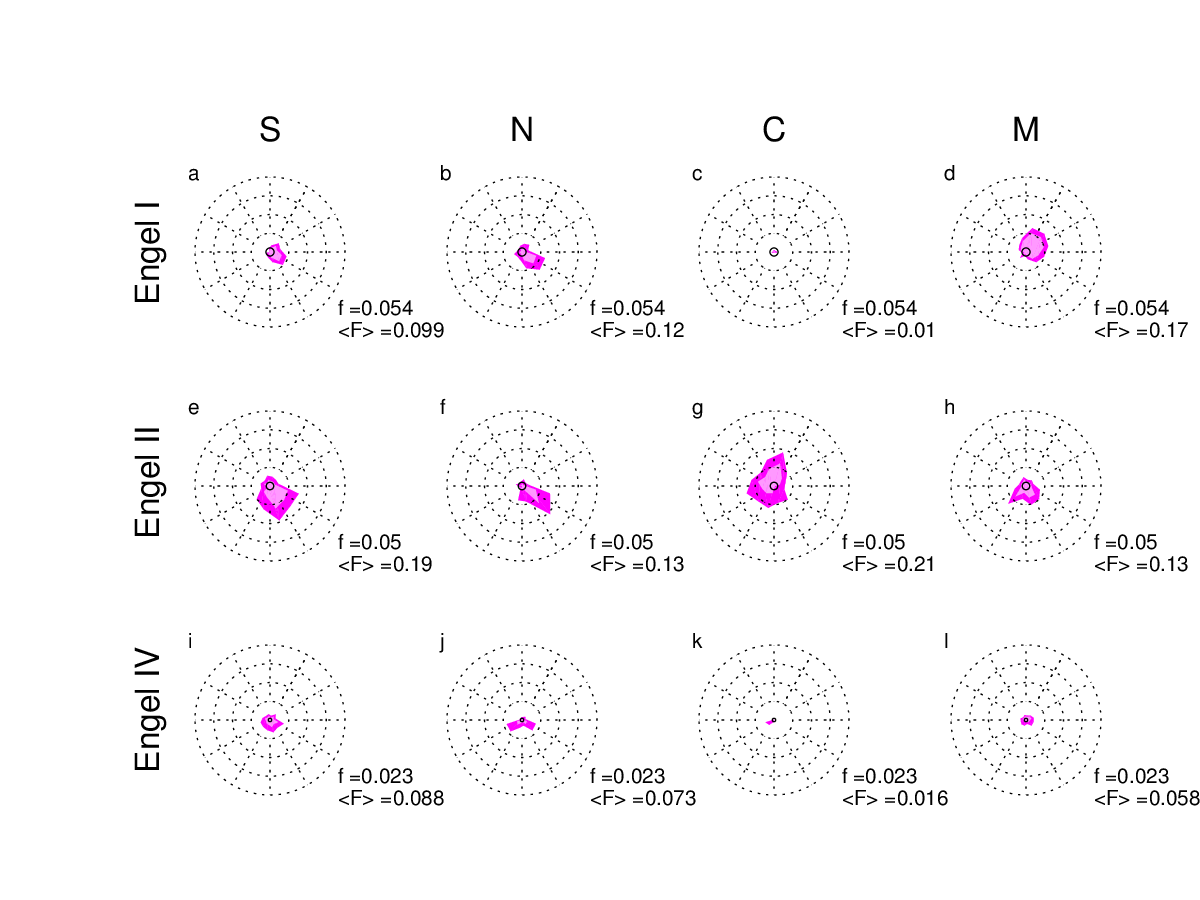

Supplement: S6 Fig — The figure arrangement is analog to Fig 5 of the main text. (TIFF) [file pone.0141023.s006.tiff]

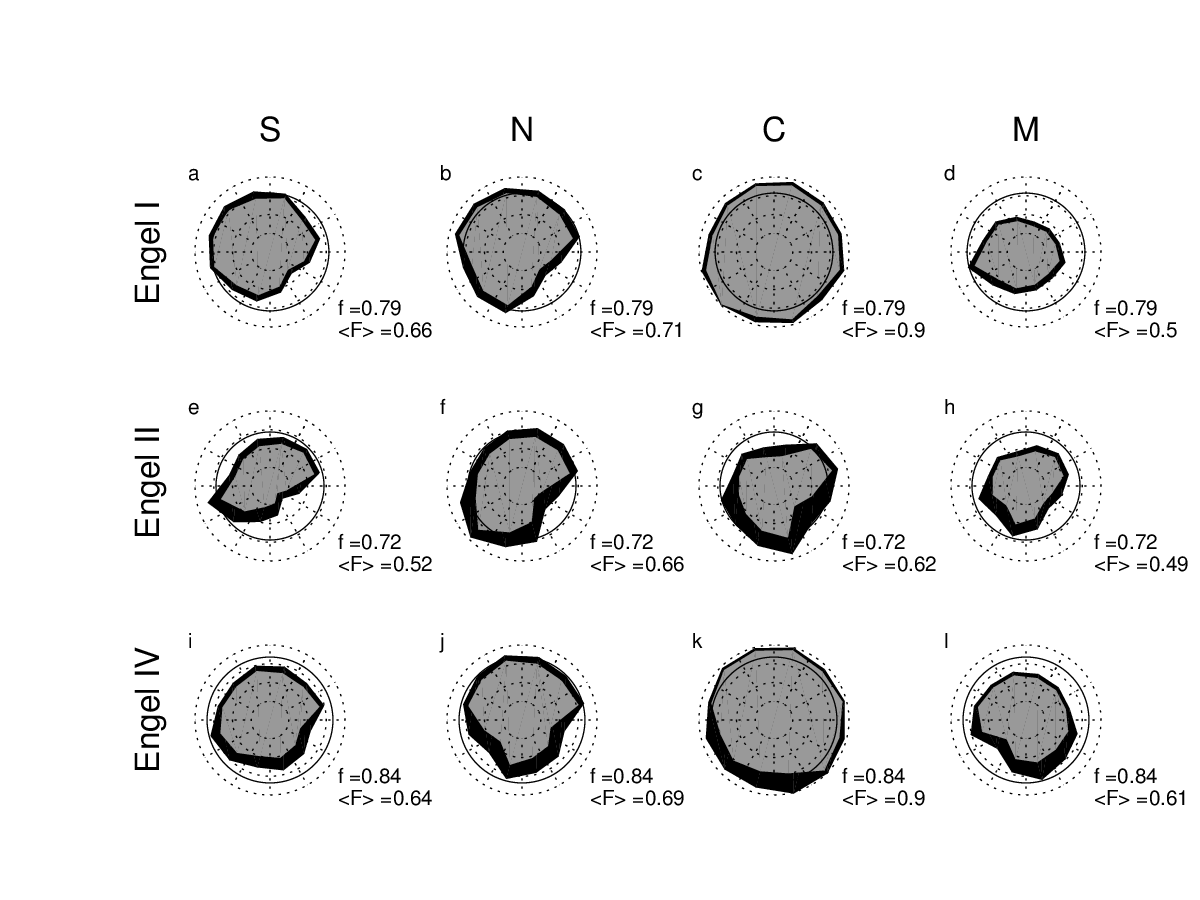

Supplement: S7 Fig — The figure arrangement is analog to Fig 5 of the main text. (TIFF) [file pone.0141023.s007.tiff]

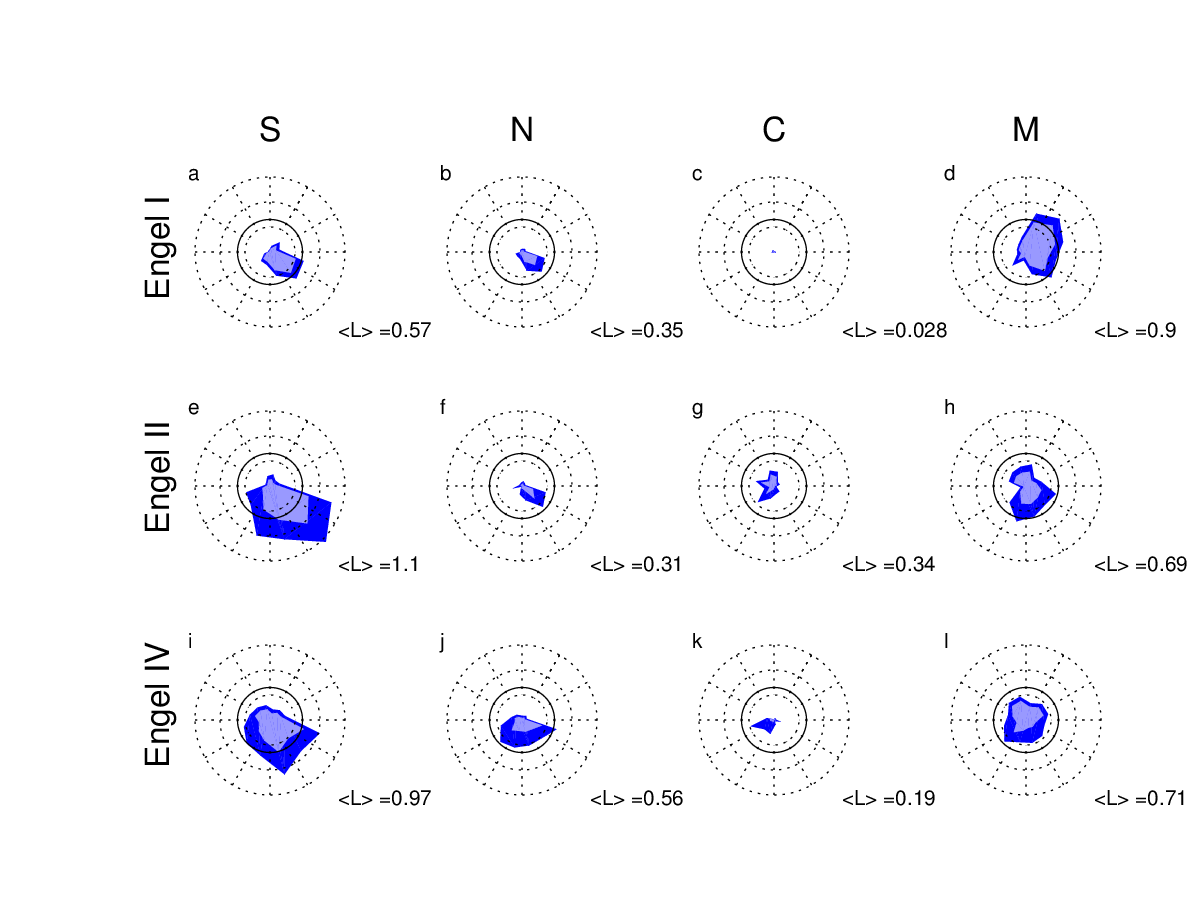

Supplement: S8 Fig — The figure arrangement is analog to Fig 6 of the main text. (TIFF) [file pone.0141023.s008.tiff]

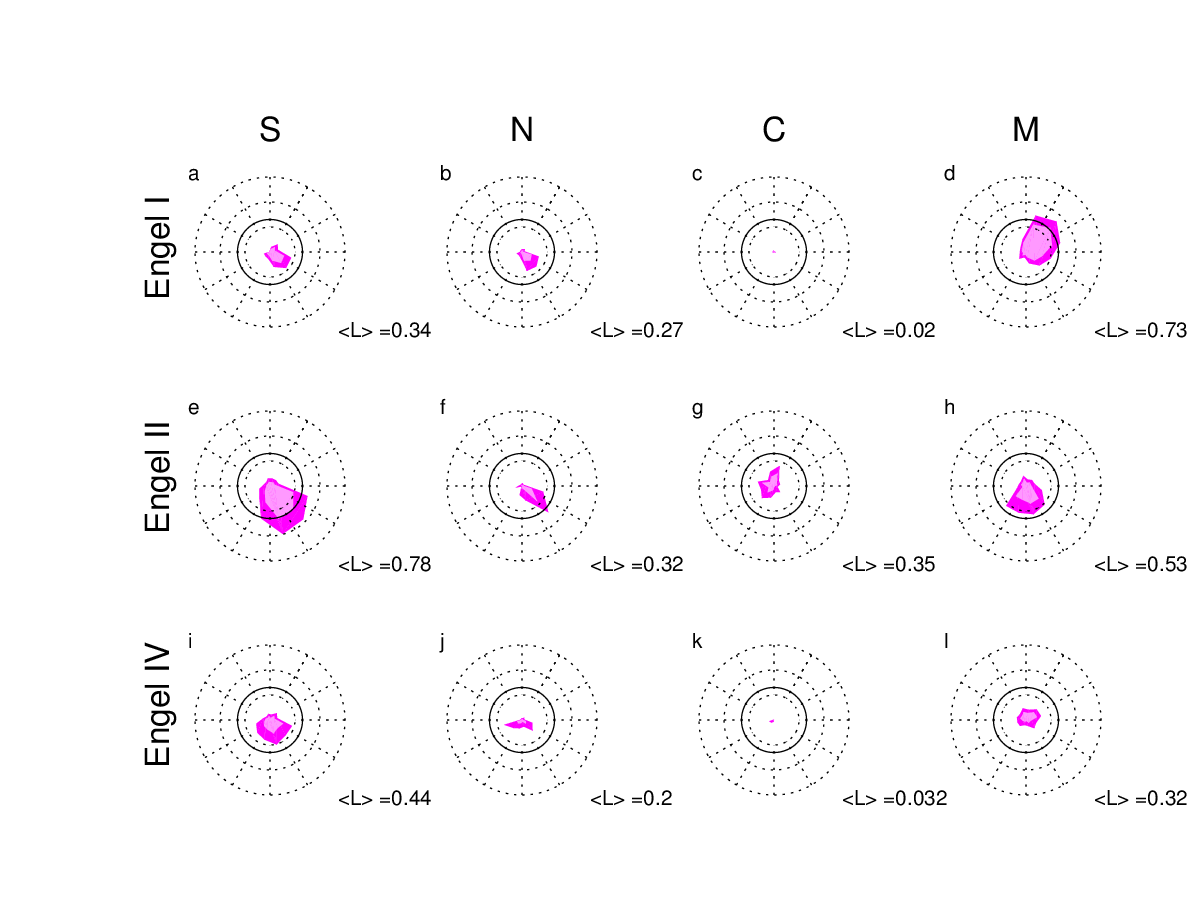

Supplement: S9 Fig — (TIFF) [file pone.0141023.s009.tiff]

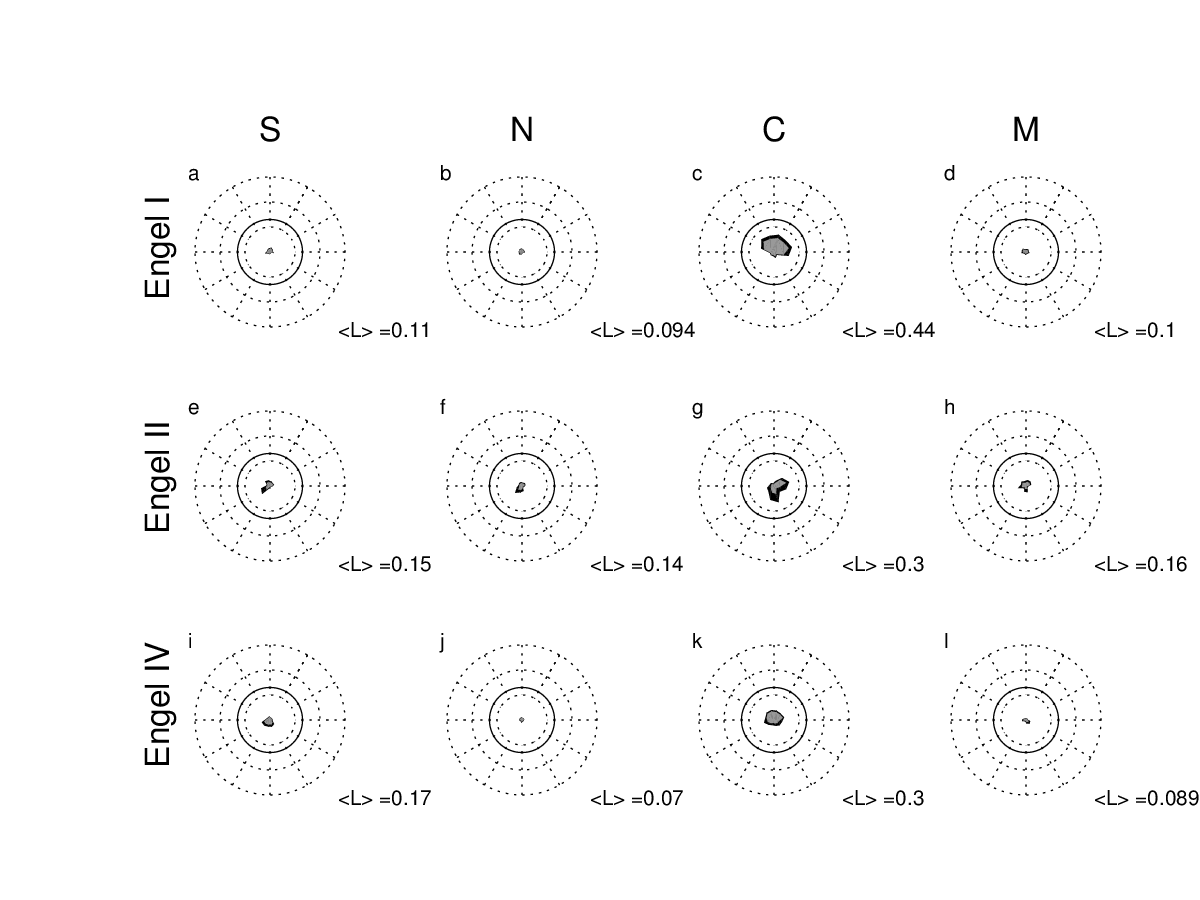

Supplement: S10 Fig — The figure arrangement is analog to Fig 6 of the main text. (TIFF) [file pone.0141023.s010.tiff]
